# A new classmate in anatomy education: 3D anatomical modeling medical students’ engagement on learning through self‐prepared anatomical models

**DOI:** 10.1002/ase.70070

**Published:** 2025-06-17

**Authors:** Muhiddin Furkan Kılıç, Afife Zehra Yurtsever, Feyza Açıkgöz, Beste Başgut, Burcu Mavi, Ezgihan Ertuç, Sinem Sevim, Tuhan Oruk, Yavuz Selim Kıyak, Tuncay Peker

**Affiliations:** ^1^ Faculty of Medicine Gazi University Ankara Turkey; ^2^ Department of Medical Education and Informatics, Faculty of Medicine Gazi University Ankara Turkey; ^3^ Department of Anatomy, Faculty of Medicine Gazi University Ankara Turkey

**Keywords:** 3D anatomical modeling, anatomy education, anxiety, curriculum, rapid prototyping, student satisfaction, virtual reality

## Abstract

Traditional education often relies on passive learning approaches, whereas modern medical students increasingly seek interactive, technology‐enhanced experiences. Despite the growing use of digital tools in anatomy education, there remains a lack of structured, student‐centered 3D modeling courses embedded within the undergraduate medical curriculum. Guided by constructivist learning theory and emphasizing student co‐creation, we designed the “Anatomical 3D Modeling Workshop”, enabling students to create, visualize, and interact with anatomical models through 3D modeling, 3D printing, and virtual reality (VR). About 29 voluntary preclinical medical students participated in a 16‐week elective course where they actively built anatomical models using professional software, produced medical animations, and experienced their creations in VR. Physical 3D prints of selected models were also produced. Outcomes were assessed through pre‐ and post‐workshop anatomy exam scores and a questionnaire. Although exam scores demonstrated a nonsignificant post‐workshop improvement, students reported substantial benefits, including stress and anxiety reduction and enhanced motivation, valuing the opportunity to co‐create educational materials. They also highlighted the workshop's contribution to their professional development. Embedding student‐centered and technology‐driven strategies into anatomy education could reduce stress and anxiety. The Anatomical 3D Modeling Workshop demonstrates the potential of integrating active, hands‐on approaches to transform medical education and better align with the expectations of learners.

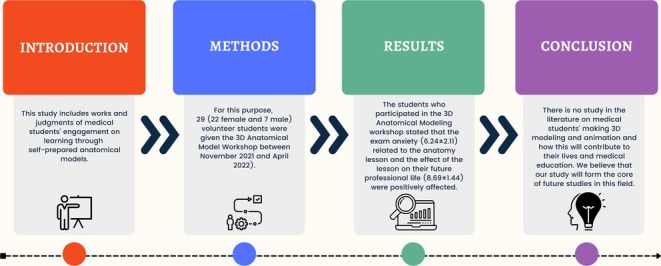

## Graphical abstract

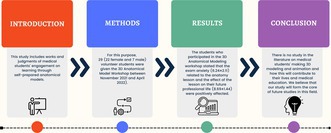

## INTRODUCTION

Learning anatomy of the human body fundamentally involves identifying its parts and understanding their three‐dimensional (3D) relationships with surrounding structures. Until the 1980s, cadaver‐based education was considered an essential component of medical and anatomical training. However, during that period, rapid advancements in computer hardware and software sparked discussions around revolutionary new technologies, such as 3D virtual modeling, virtual reality (VR), and augmented reality (AR).^1^ As a result, these technologies have become more affordable and increasingly integrated into our daily lives, which is reflected in research. For example, a study^2^ reported finding 21,667 entries related to VR and 9944 related to AR in the Web of Science Core Collection database.

3D Computer‐Generated Imagery (CGI) typically appeals to large audiences through its use in popular entertainment and art. The earliest known example is “A Computer Animated Hand” by Edwin Catmull and Fred Parke in 1972. In this project, Catmull drew 350 triangles and polygons on his own hand using ink, and by carefully animating this data, he created a 3D animation using software he programmed himself.^3^ However, the use of 3D CGI in medicine has been relatively limited. This is primarily due to the necessity of using real human CT or MRI data, which must be based on accurate anatomical, physiological, and clinical assessments.^4^, ^5^ Moreover, the ethical considerations related to the use of real human imaging data must be rigorously observed to ensure privacy and consent. Additionally, creating these models requires individuals who are both medically knowledgeable and skilled in 3D modeling.

3D medical computer models allow users to change perspectives, toggle between normal anatomy and pathological conditions, and observe the variability of the human body. These models are also reusable, portable, durable, and consistent.^6^ As a result, they can be accessed beyond the conventional boundaries of lecture halls and laboratories. This flexibility has led anatomy educators to recognize the pedagogical potential of virtual modeling. Moreover, educators view these computer models as evolving and practical tools—sometimes even as viable substitutes for traditional educational materials.^7^ Consequently, they recommended integrating these digital representations into medical curricula to enhance the understanding of complex spatial and 3D information.^6^ Additionally, their printed versions also provide realism that reinforces anatomical learning, offering another reusable alternative to traditional cadaver‐based instruction.^7^ Related research showed that 3D‐printed models significantly enhance anatomy education by improving test scores, accuracy, response time, and student satisfaction compared with traditional methods such as cadavers and 2D images.^8^, ^9^, ^10^, ^11^

Medical educators often critique medical and anatomical education based on their professional experiences and aim to improve it by reflecting on those insights. However, the perspectives of medical students regarding these subjects often differ from those of educators.^12^ For example, anatomy educators frequently critique the limited time allocated to anatomy within medical curricula, whereas students often prioritize aspects such as patient or cadaver contact and self‐directed learning, reflecting a divergence in perceived curricular needs and educational priorities.^12^ The key lies in bringing educators and students together on a common ground to ease the challenges of medical education, make it more engaging for students, and ultimately improve learning outcomes. Therefore, a previous study offered undergraduate student involvement in 3D modeling.^13^ Another study involved them with some limited participation in instructor‐led modeling of a single organ.^14^ The evaluation in the study was purely quantitative and lacked aspects of student motivation, emotional impact, long‐term engagement, or the integration of VR and 3D printing. It is an important gap considering that feelings of anxiety and fear of failure are common and can hinder learning in anatomy education, as seen in the phenomenon of neurophobia reflected in studies on neuroanatomy.^15^ Taking these factors into account is essential, particularly within a constructivist learning theory,^16^ which emphasizes that learners build knowledge more effectively through active engagement.

Since 2007, our faculty has been producing educational materials incorporating 3D medical modeling and animations. These resources have been integrated not only into anatomy education but also into related disciplines such as histology and embryology. In parallel with educational development, our faculty has also engaged in research on the integration of 3D technologies into anatomy teaching.^17^ Building upon this foundation and in response to student demand, and also to address the gap mentioned above, we launched the “Anatomical 3D Modeling Workshop”. Compared with the previous studies, we conducted a broader, student‐centered, workshop‐style course where undergraduate medical students created their own 3D models, engaged with immersive technologies, and reflected on their learning experiences. The research questions of our study are as follows:

1. Does participation in a student‐centered 3D anatomical modeling workshop affect students’ academic performance in anatomy exams?
2. How do students perceive the workshop's impact on their stress and anxiety levels, engagement, and the educational value of learning 3D modeling?

## MATERIALS AND METHODS

**FIGURE 1:**
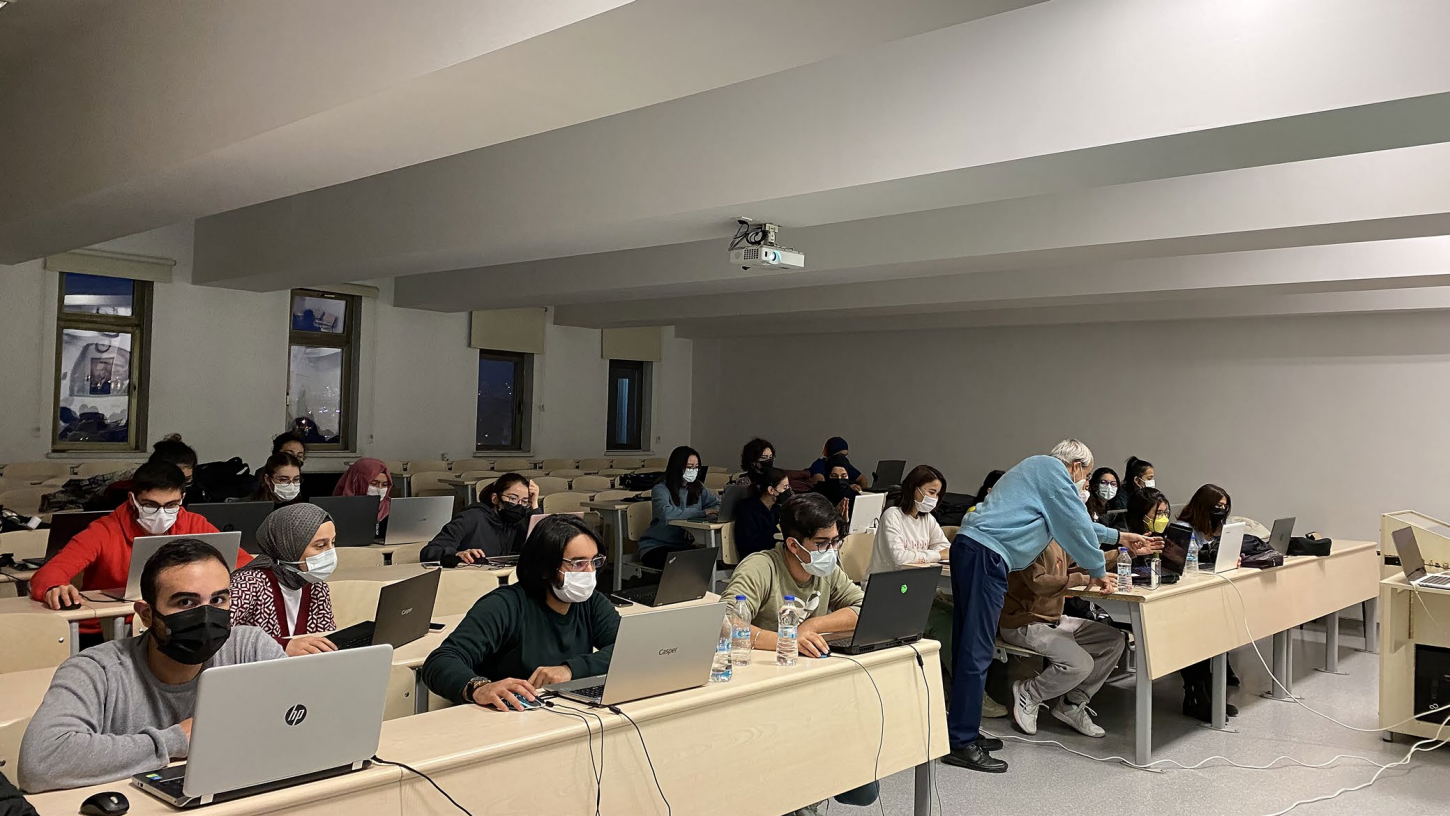
A picture from the anatomical modeling workshop elective course.

The workshop took place between November 30, 2021, and April 5, 2022 (Table 1). It was conducted with the voluntary participation of 29 medical students from the 1st, 2nd, and 3rd years of study at our institution (Figure 1), including 7 males (24.1%) and 22 females (75.9%). The majority of participants were second‐year medical students, accounting for 72.4% (*n* = 21), followed by first‐year students at 24.1% (*n* = 7), and one third‐year student representing 3.4% of the total. This distribution indicates a predominant interest from students in the early preclinical phase, particularly those in their second year of medical education. Students were informed and recruited through official announcements made via the university's announcement system. Additionally, faculty members shared information about the study during relevant anatomy lectures and practical sessions. Interested students were invited to participate voluntarily, and informed consent was obtained prior to inclusion. Official approval for the workshop and the protocol was granted by the medical faculty administration, and the study protocol received approval from the Gazi University, Faculty of Medicine (No: 1205565).

**TABLE 1:** The schedule of the first 3D medical modeling and animation curriculum for undergraduate medical students in the world.

Anatomy workshop curriculum			
Class	Date	Subject	Content
1	11/30/2021	Introduction	General information on medical modeling
2	12/7/2021	Introduction‐2	Continuing general information on medical modeling
3	12/14/2021	Modeling of the erythrocyte	Learning how to see the shape created in the modeling program from different angles and how to change its position Learning basic colorization Learning how to use subdivision surface, bevel, extrude, and inset tools
4	12/21/2021	Modeling of the vessel	Learning how to merge projects Learning how to change the location and multiplication of erythrocytes using cloner and random tools
5	1/4/2021	Modeling of the diapedesis	Learning the function and how to use the displacer tool Learning how to use the bulge tool and using the spline to give the new cell a movement
6	1/11/2021	Modeling of the cell apoptosis	Learning the function of cloth and cloth collider animation tags on the spheres
7	1/18/2021	Modeling of the ciliary epithelium and the neuron	Learning how to use the function of adding hair to form cilia‐like structures Learning how to give movement to hair extensions by using the turbulence tool
8	2/8/2022	Modeling of the intra‐abdominal organs	Learning the function of the camera tool
9	2/15/2022	Modeling of the phagocytosis of bacteria	Learning how to use copy shader and paste shader functions Learning the function of the protection tag and how to use the camera tool
10	2/22/2022	Modeling of the cataract surgery	Learning how to use the magnet and pose morph tools
11	3/1/2022	Modeling of the passage of nutrients from the stomach	Learning how to use the volume builder and volume mesher tools
12	3/8/2022	Modeling of the passage of nutrients from the stomach‐2	Repeating the modeling of the passage of nutrients from the stomach
13	3/15/2022	Modeling of the angioplasty	Learning how to use the pose morph animation tag
14	3/22/2022	Modeling of the angioplasty‐2	Continuing the modeling of the angioplasty
15	3/29/2022	Modeling of the uterus and the adnexa	Learning how to use the lathe tool Learning how to use the volume mesher, volume builder and subdivision surface tools
16	4/5/2022	Modeling of the uterus and the adnexa‐2	Continuing the modeling of the uterus

This study is informed by constructivist learning theory,^16^ which posits that learners build knowledge through active engagement rather than passive receiving. More specifically, involving learners in the co‐creation of learning experiences can further enhance engagement, ownership, and motivation,^18^ which are factors critical for effective learning.

The workshop was initiated at the direct request of students, who expressed a desire not only to engage with new‐generation educational technologies but also to learn how to create anatomical and functional 3D virtual models themselves. Their goal was to assess whether these self‐produced models would enhance their understanding of anatomy and contribute meaningfully to their medical education. To maintain high levels of engagement and motivation, weekly topics were selected in alignment with both the medical curriculum and the students’ preferences. This student‐driven approach led to the development of a structured and progressive 16‐week‐long curriculum, which is notable for being one of the first 3D medical modeling curricula in the world designed specifically for undergraduate medical students.

**FIGURE 2:**
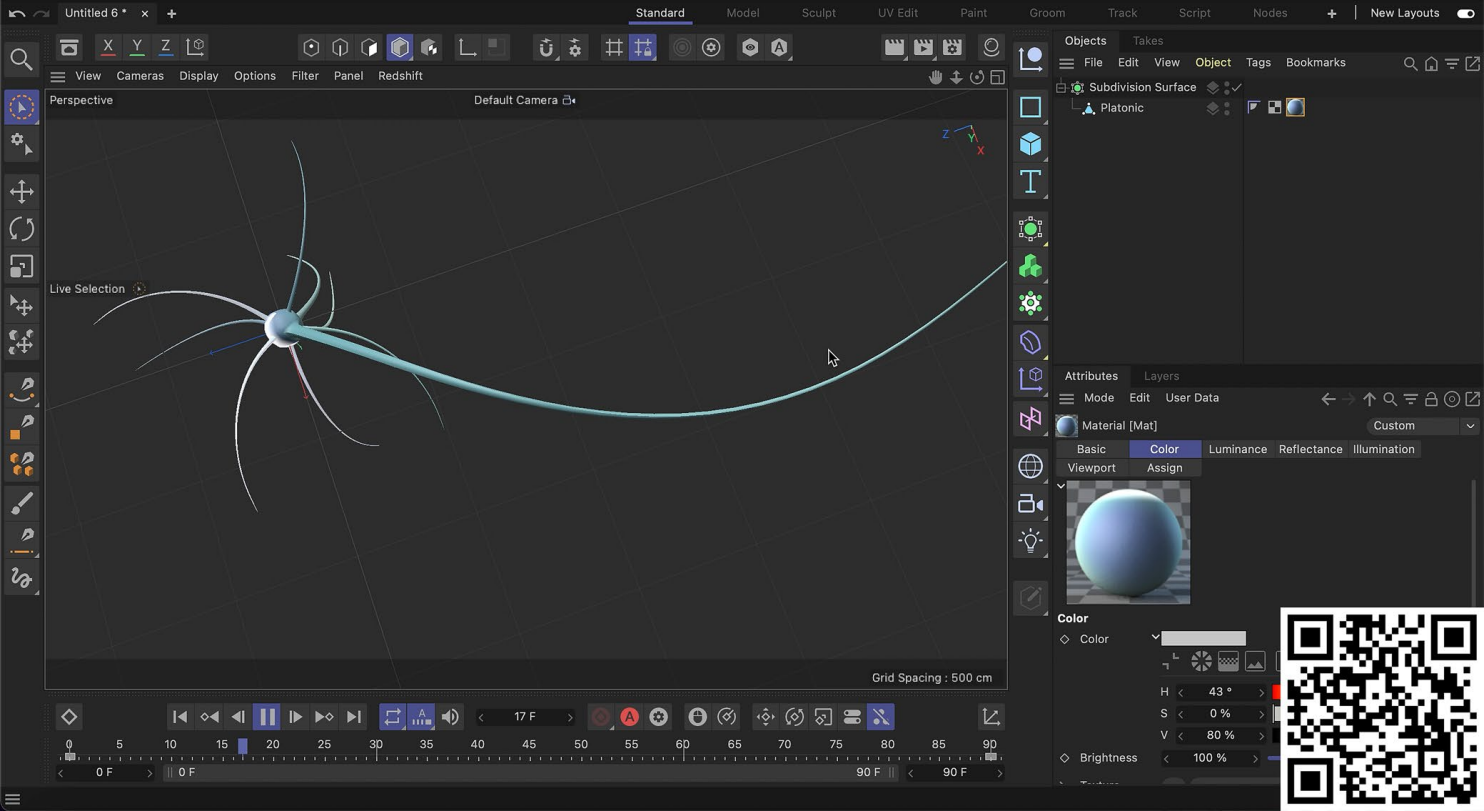
An education material created by the lecturer. (You can watch the video via the QR code.)

Students created their anatomical models using Cinema 4D (Maxon Computer, Friedrichsdorf, Germany), a professional 3D modeling and animation program that they installed on their own computers. In preparation for each lesson, the instructor produced and shared detailed video tutorials (Figure 2) via the web, demonstrating the construction of the specific anatomical models to be created that week. The weekly sessions were conducted in person every Tuesday from 5:00 to 7:00 p.m., during which students reviewed their progress, addressed challenges, and received individualized feedback from the expert. The anatomical correctness of the models was verified using textbooks and atlases, which were also used to select accurate textures and colors for the final models.

**FIGURE 3:**
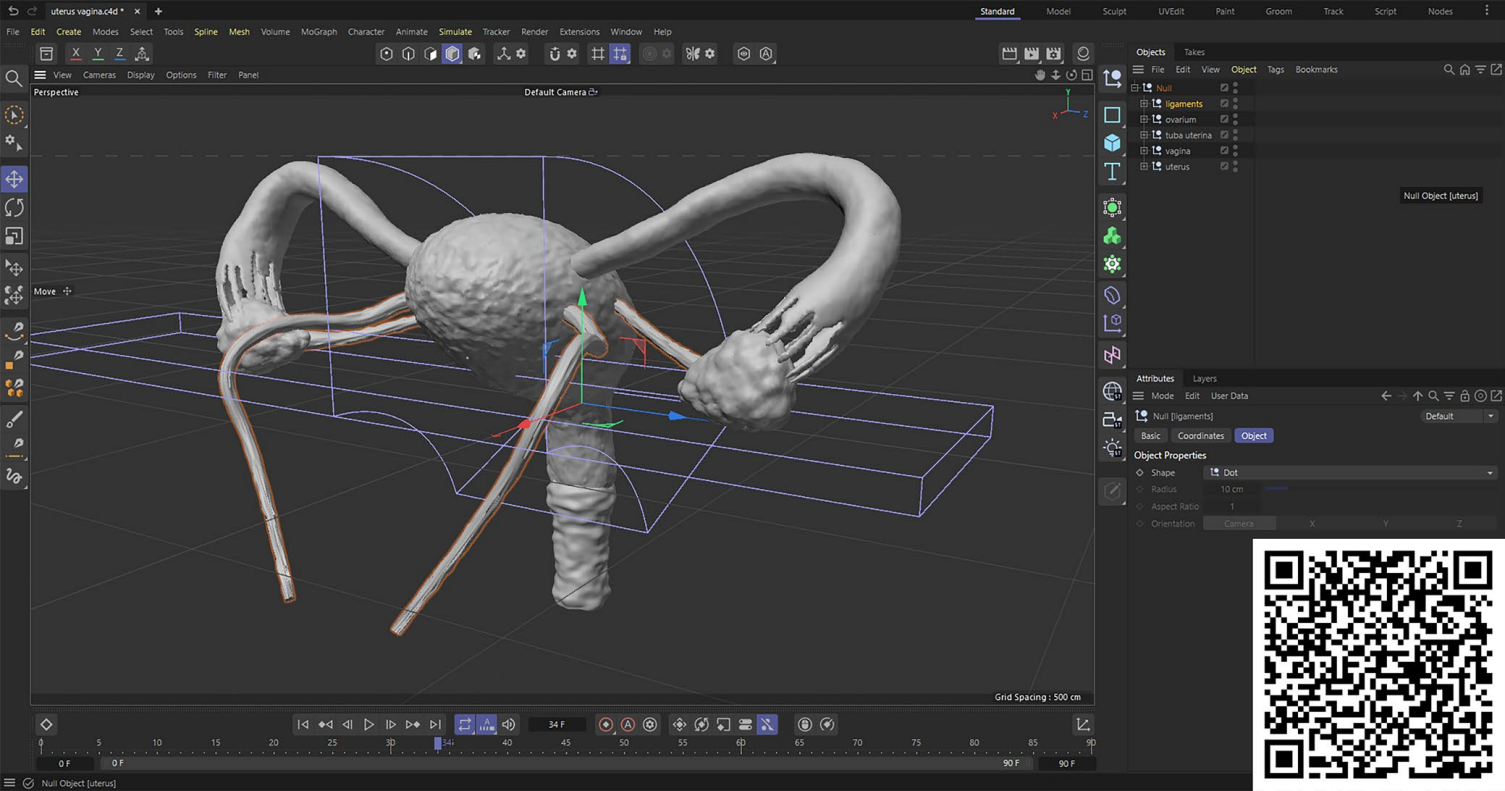
Some of the instruction videos have been shown in the playlist. (You can watch the videos via the QR code.)

For each week, every model was determined according to the topic, but the difficulty of the models was chosen from basic to tough. For example, students first modeled simple structures like erythrocytes and vessels to learn basic tools, then advanced to complex anatomical systems such as intra‐abdominal organs, cataract surgery, and angioplasty, addressing both the challenges of learning anatomy and mastering 3D modeling techniques. Initially, the goal was to teach students how to use the program and create a basic structure. Students then began to learn how to make and animate increasingly intricate models. For example, students learned to model a ciliary epithelium and a neuron so that they were able to create a model of angioplasty. To facilitate the learning process, before the lesson every week, a preparation video has been created and shared with students (Supporting Information S1). Some of the instruction videos, including those on the modeling of the ciliary epithelium, neuron, duodenum, pancreas, gallbladder, cataract surgery, angioplasty, and uterus, have been shown in the playlist (Figure 3).

**FIGURE 4:**
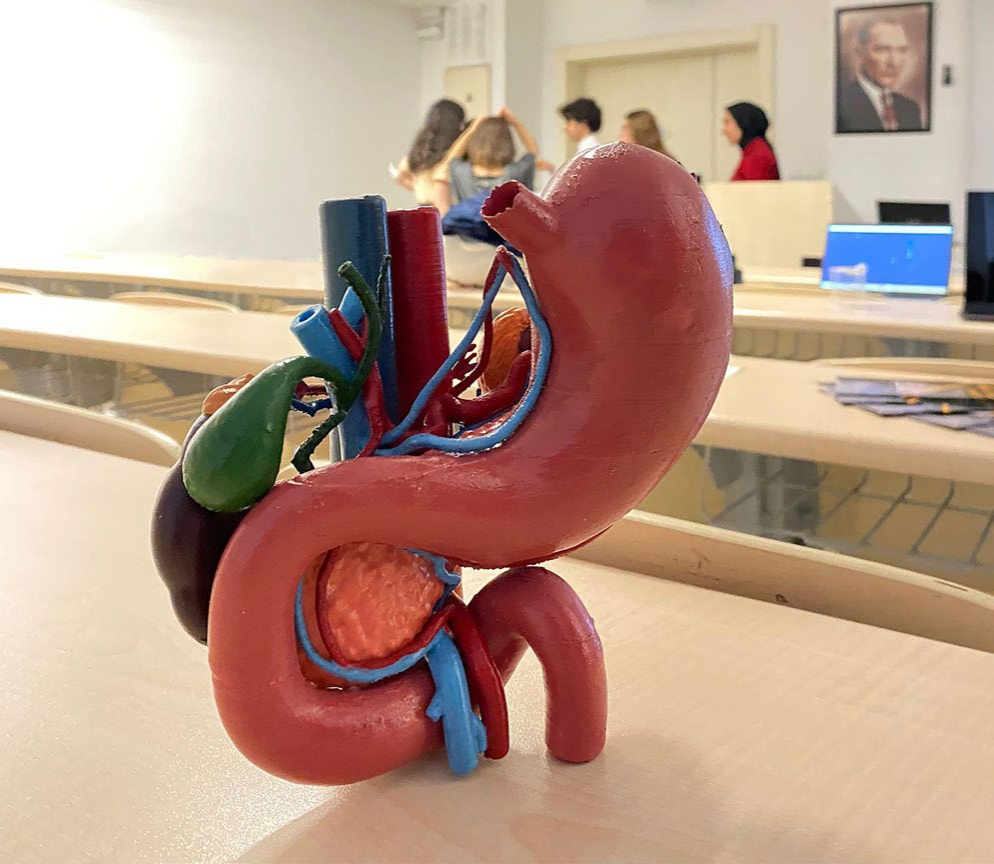
A sample of 3D‐printed and painted models that were created by the students.

After digital modeling was completed, physical 3D prints were produced by selecting the best student models. These prints were painted using model paint to visually enhance anatomical structures based on conventions found in anatomy atlases (Figure 4). Each student used ~121 g of *z*‐abs plastic filament for their 3D‐printed model, and each printing session took around 35 h. A cost analysis revealed that the average expense per printed model was $5.90, making the process both accessible and sustainable for our medical school, particularly given the limited financial resources.

**FIGURE 5:**
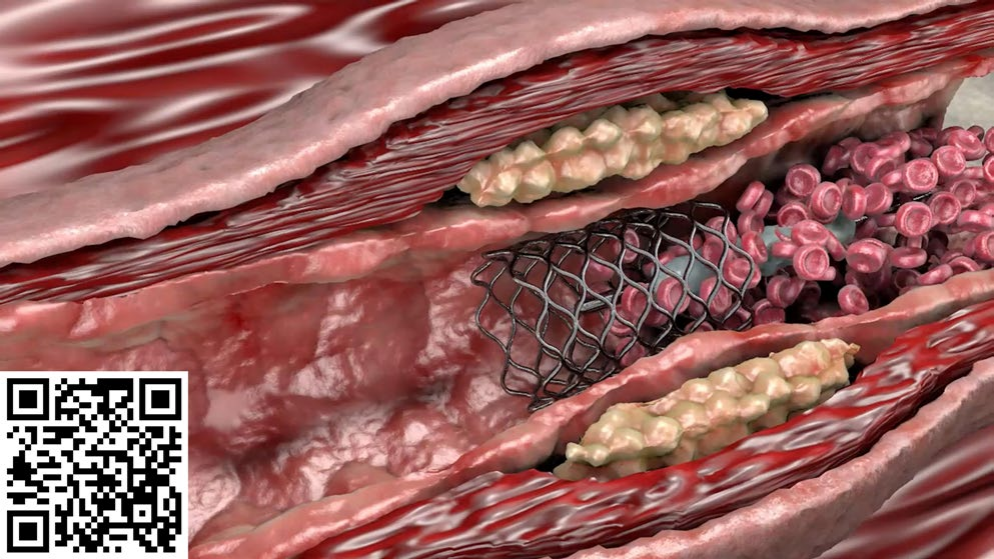
A 3D model and the related animation. (You can watch the animation via the QR code.)

**FIGURE 6:**
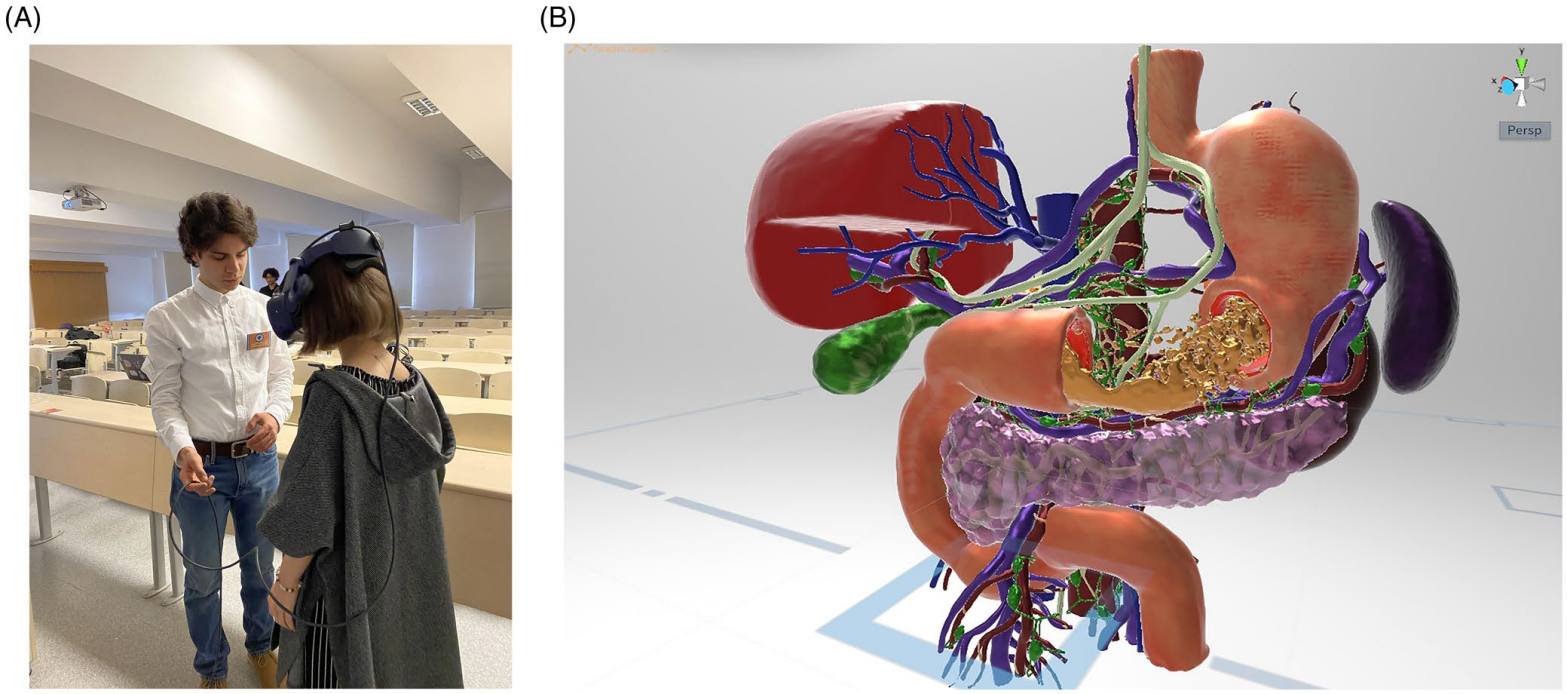
(A, B) Images of the virtual reality application of the models created by the students.

Students also developed related medical animations (Figure 5), providing dynamic visualizations of the modeled structures. Furthermore, the completed models were experienced in VR using RoT VR equipment provided by infoTRON (Ankara, Turkey). Students were able to walk around and explore their models in immersive 3D space, adding another dimension to their learning (Figure 6A,B).

**FIGURE 7:**
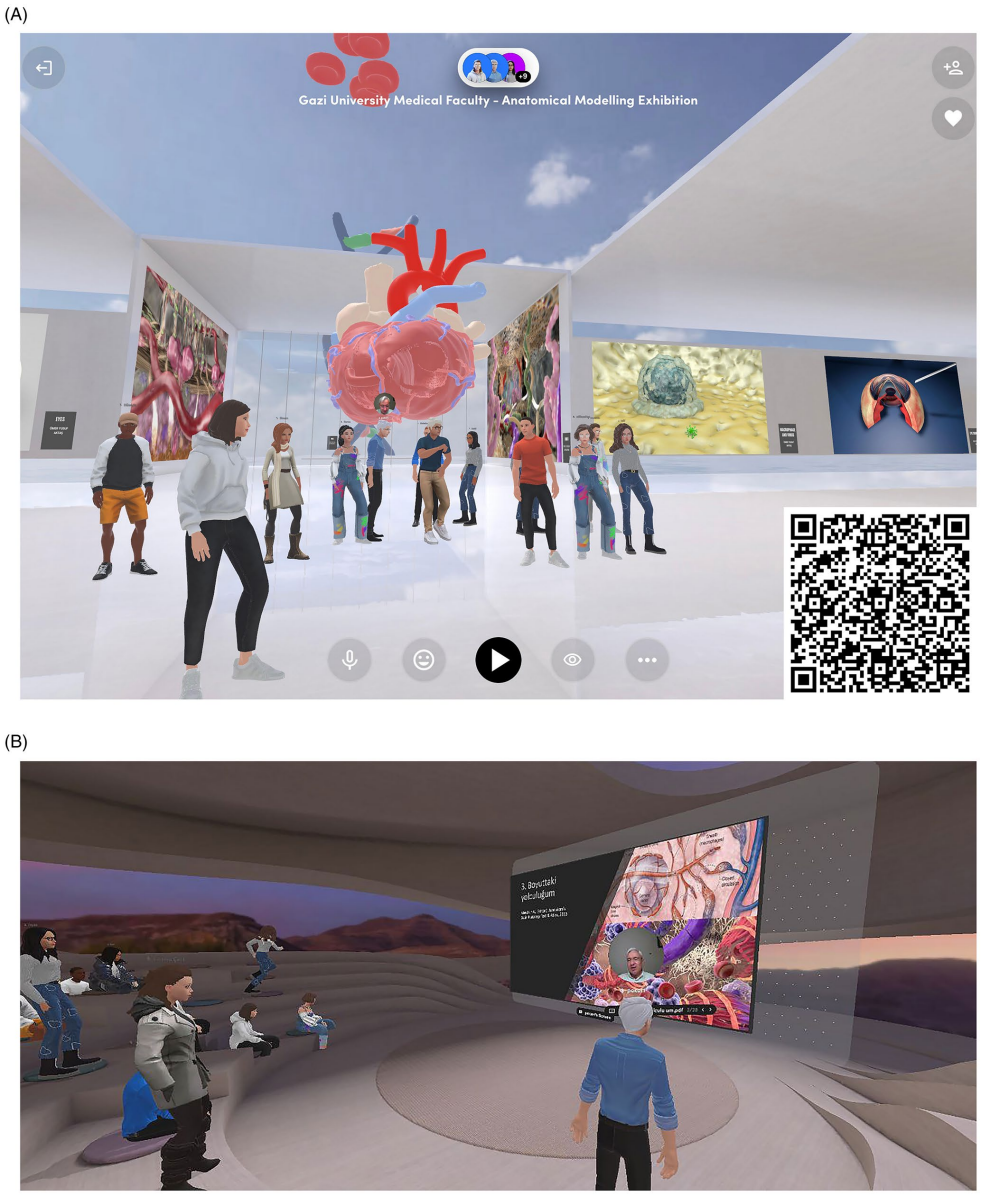
(A, B) Images of the virtual exhibition and conference on Spatial.io. (You can access the virtual exhibition via the QR code.)

Students who successfully completed the 16‐week course received certificates of achievement, recognizing their commitment and accomplishments. To showcase the models and animations created during the workshop, an online virtual exhibition was curated using Spatial.io, a platform that allows for immersive exploration of digital content. The exhibition was made accessible via QR code, allowing viewers to engage with the models in a VR environment (Figure 7A,B). Additionally, the booklet was physically and virtually prepared and shared in the faculty (Supporting Information S2). This exhibition served not only to celebrate student achievements but also reinforced the study's aim of enhancing engagement, motivation, and applied anatomical understanding through interactive, technology‐driven experiences.

### Data collection

During the research process examining the effects of the modeling workshop on anatomy performance, students’ exam grades were collected via Google Forms (later confirmed with official documentation). Upon completion of the workshop, an anonymous questionnaire was administered to all participants to evaluate their experiences. The questionnaire consisted of two parts: nine rating‐scale questions (scored from 1 to 10) covering aspects, such as content quality, coordination with the instructor, impact on medical lecture comprehension, stress and anxiety reduction, interest in 3D printing, and future applicability, along with one open‐ended question. The questionnaire items were designed to evaluate the workshop's impact on students’ academic performance, emotional well‐being, engagement with 3D technologies, and perceptions of instructional quality.

### Statistical analysis

As only second‐year students had anatomy exams both before and after the workshop, the main statistical analysis of the exam scores was conducted on this group. Because the number of questions varied between exams, all scores were normalized by calculating the percentage of correct answers. Among second‐year students, Exam 1 and Exam 2 occurred before the workshop, and Exam 3 and Exam 4 followed it. A total of 21 students were included in the statistical analysis of the exam performance. However, ratings of the anatomical modeling workshop included all participants’ input.

Data were analyzed using the Statistical Package for the Social Sciences (SPSS) for Windows, version 23.0 (SPSS Inc., Chicago, USA). Descriptive statistics were presented as mean ± standard deviation and median (minimum–maximum) for continuous variables, and as frequencies and percentages for categorical variables. The normality of data distribution was assessed using visual methods (e.g., histograms), central tendency measures (mean and median), and formal tests including the Kolmogorov–Smirnov and Shapiro–Wilk tests, along with skewness, kurtosis, and coefficient of variation values. Nonparametric tests were performed where assumptions of normality were not met. Specifically, the Wilcoxon signed‐rank test and the Friedman test were used. A *p*‐value of < 0.05 was considered statistically significant.

In addition to the quantitative analysis, qualitative analysis of responses to the open‐ended questionnaire item was carried out by the research team, drawing on elements of the thematic analysis method.^19^ After familiarizing ourselves with the open‐ended responses, we performed initial coding to identify key points. Similar codes were grouped to form broader categories. Through iterative review and refinement, we structured the findings into two main types of themes: problems experienced by students during the workshop and the corresponding solutions they proposed. This problem–solution framework was used to organize and present the qualitative results.

## FINDINGS

### Anatomy exam performance

As presented in Table 2, the average post‐workshop exam scores (Exam 3 and Exam 4) of second‐year students who participated in the Anatomical 3D Modeling Workshop were higher than their pre‐workshop scores (Exam 1 and Exam 2). However, this increase was not statistically significant (*p* > 0.05). Further analysis compared the average exam scores of second‐year students based on gender. The results indicated that the difference in exam performance between male and female students was not statistically significant (*p* > 0.05).

**TABLE 2:** Anatomy exam scores of 2nd year medical students (*n* = 21).

	Mean ± SD	Median (min–max)	*p*
Exam 1 (Neurological system)	63.49 ± 19.22	70.83 (29.17–91,67)	0.829*
Exam 2 (Respiratory and cardiovascular systems)	62.20 ± 17.28	62.50 (18.75–87.50)	
Exam 3 (Digestive system and metabolism)	65.87 ± 15.03	66.66 (33.33–94.44)	
Exam 4 (Endocrine and urogenital system)	69.04 ± 16.06	66.66 (41.67–100.00)	

* Friedman test.

### Questionnaire

Students were asked to rate the workshop's effectiveness in reducing their stress and anxiety related to medical education on a scale from 1 to 10, where 1 indicated “no change” and 10 indicated “complete change.” The results presented in Table 3 showed a moderate overall reduction in stress levels (6.24 ± 2.11). In addition to this, students reported that the applicability of the workshop content to their future professional life (8.69 ± 1.44) increased their academic engagement and motivation. A strong interest was observed in the hands‐on aspects of the course, particularly in obtaining 3D‐printed versions of their models (9.45 ± 1.18). Students showed enthusiasm for painting their prints with realistic anatomical features, mirroring those seen in professional atlases.

**TABLE 3:** Participant ratings of the anatomical modeling workshop based on a 1 (low)–10 (high) scale.

Evaluation aspect	Mean ± SD	Median (min–max)
Rating for content	8.24 ± 1.35	8.00 (5–10)
Contribution of the workshop to medical lectures	5.66 ± 2.25	6.00 (2–10)
Recalling the features of the program used after the workshop	6.90 ± 1.95	7.00 (2–10)
Request for 3D printout from the 3D modeled structure	9.45 ± 1.18	10.00 (6–10)
Rate of reduction of stress and anxiety toward classes	6.24 ± 2.11	7.00 (1–10)
Rate of using workshop knowledge in future professional life	8.69 ± 1.44	9.00 (5–10)
Coordination of students with the instructor	7.66 ± 1.95	7.00 (3–10)
Adequacy of the instructor's knowledge	9.48 ± 0.78	10.00 (7–10)
The instructor's explanation is clear and understandable	9.07 ± 1.06	9.00 (7–10)

The open‐ended questionnaire responses provided insights into the challenges encountered during the workshop and included student‐suggested solutions. Four themes were found:

Problem 1: Although the instructor's explanations were generally rated as clear, some students reported difficulty following the lessons in real‐time, which affected their ability to stay synchronized with the rest of the class.
Solution: The availability of video recordings prior to class helped mitigate this issue, allowing students to review the material at their own pace.
Problem 2: Classroom overcrowding made it challenging for the instructor to provide individual support, causing delays for students who needed additional help.
Solution: Dividing the class into smaller working groups was suggested to enhance instructor–student interaction and improve overall efficiency.
Problem 3: Technical inconsistencies—including differences in software versions, computer hardware, and classroom infrastructure—not only disrupted the flow of lessons but also created difficulties in viewing the projected screen and using equipment effectively.
Solution: Conducting the course in dedicated computer labs with standardized hardware and optimized visibility was recommended to overcome these logistical challenges.
Problem 4: Some students lacked familiarity with the 3D modeling tools at the beginning of the course, which slowed down their progress.
Solution: Students proposed allocating the first 2 weeks of the workshop to learning and practicing the basic functions of the modeling software before starting anatomical modeling tasks.

## DISCUSSION

This study aimed to evaluate the educational value of a student‐centered 3D anatomical modeling workshop, focusing not only on students’ academic performance but also on their broader educational experience such as emotional response and engagement. While the quantitative results showed no statistically significant improvement in anatomy exam scores, qualitative feedback and engagement metrics support the potential of such workshops in anatomy education.

Our findings align with previous literature acknowledging the complexity of learning human anatomy and the increasing relevance of technology‐enhanced learning tools. Although earlier studies^14^ demonstrated a significant increase in short‐term exam performance through instructor‐led 3D modeling of a single organ, their scope was limited. Student motivation and stress reduction are important aspects to consider. It is also important to incorporate further involvements such as VR, 3D printing, and model painting. Therefore, our workshop provided a more comprehensive and immersive learning opportunity in which students actively created different anatomical models, explored them in VR, and engaged with feedback in a structured yet flexible setting.

The lack of statistically significant improvement in exam performance, particularly among second‐year students, may be attributed to the workshop's focus on 3D modeling skills and spatial reasoning rather than direct exam preparation. Anatomy education in medical school is broad, detailed, and often system‐based; thus, a 32‐h elective course cannot cover the same volume or depth as the core curriculum. Nevertheless, the observed increase in average post‐workshop exam scores, albeit not significant, is an indicator that such interventions may contribute positively to academic performance over time, especially if better integrated into the formal curriculum.

More notably, the qualitative data reveal important benefits of the workshop beyond test scores. Students reported a moderate reduction in stress and anxiety levels. Although this is not a direct intervention for mental health, the integration of innovative teaching methods, such as 3D anatomical models and immersive technologies, has been shown to enhance medical students’ learning experiences. These approaches not only improve knowledge acquisition but also increase student engagement and satisfaction, which are critical factors in supporting mental well‐being during the rigorous preclinical years of medical education.^20^ Similar findings have been reported in studies where students work with clay models, noting positive results such as increased engagement associated with learning anatomical structures.^21^, ^22^, ^23^ The benefit may also be influenced by the painting component in the workshop; similar to our findings, a study^24^ reported that while the use of color in body painting did not significantly impact knowledge retention, students found the activity enjoyable. We also observed a strong sense of motivation among the students. Their high enthusiasm for printing and painting 3D‐printed models suggests a deepened engagement with anatomical content, transforming learning from passive reception to active exploration. This supports the notion that when students are given ownership over their learning tools and processes, their educational experiences become more meaningful.^18^

The integration of VR in the workshop also reflects current trends in digital anatomy education.^25^ Literature suggests that virtual models can enhance spatial understanding, especially for complex anatomical relationships.^26^ In our study, VR allowed students to interact with their own digital creations in an immersive way, further bridging the gap between theoretical knowledge and applied anatomical visualization. This aspect of the workshop was particularly well received and should be expanded in future implementations.

The challenges identified through the open‐ended responses—such as technical difficulties, classroom overcrowding, and the steep learning curve of the modeling software—are valid concerns and highlight the importance of proper infrastructure and preparation in delivering such workshops. Students themselves proposed practical solutions, including conducting sessions in computer‐equipped classrooms and allocating dedicated time at the beginning of the course for tool familiarization. These suggestions will inform future refinements to the curriculum and delivery method.

While this study provides valuable insights into the educational potential of 3D anatomical modeling workshops, several limitations should be acknowledged. First, the relatively small sample size and single‐institution setting limit the generalizability of the findings. Additionally, the statistical analysis of exam performance was confined to second‐year students, as only they had comparable pre‐ and post‐workshop exam data. Furthermore, the anatomy exams used may not have been designed to fully capture spatial learning outcomes, potentially underrepresenting the specific benefits of the workshop. Another limitation is that the absence of a control group and the reliance on self‐reported data for questionnaire responses may introduce bias and restrict causal interpretation.

Despite these limitations, the study opens several promising avenues for future research and curriculum development. Expanding the workshop to include a larger and more diverse student population across multiple institutions would help validate and refine its educational impact. Incorporating a control group or randomized design could further strengthen the evidence base. In terms of content, future iterations of the course should dedicate more structured time at the beginning for software training and be delivered in appropriately equipped computer labs to minimize technical barriers. Longitudinal studies are also needed to assess whether the skills and insights gained through 3D modeling have lasting effects on students' academic performance, clinical reasoning, and professional practice. Furthermore, integrating similar workshops into the formal medical curriculum, rather than as electives, may enhance accessibility and ensure broader student engagement.

## CONCLUSIONS

As conclusion, the Anatomical 3D Modeling Workshop demonstrated that student‐centered, technology‐enhanced learning can enrich the anatomy education experience beyond traditional methods. While no statistically significant improvement was observed in exam performance, the workshop positively influenced students' engagement, motivation, and interest in applying anatomical knowledge through hands‐on and immersive tools such as 3D modeling, printing, and VR. These findings highlight the importance of integrating interactive and creative approaches into medical curricula, not only to support spatial understanding but also to foster long‐term professional skills. As medical education continues to evolve, initiatives like this workshop represent meaningful steps toward aligning educational practices with the needs and expectations of the next generation of healthcare professionals.

## AUTHOR CONTRIBUTIONS

**Muhiddin Furkan Kılıç:** Conceptualization; data curation; investigation; methodology; project administration; resources; visualization; writing – original draft; writing – review and editing. **Afife Zehra Yurtsever:** Resources; writing – original draft; writing – review and editing. **Feyza Açıkgöz:** Data curation; resources; software; visualization; writing – original draft. **Beste Başgut:** Resources; writing – review and editing. **Burcu Mavi:** Conceptualization; resources; writing – original draft; writing – review and editing. **Ezgihan Ertuç:** Conceptualization; resources; writing – original draft. **Sinem Sevim:** Resources; writing – original draft; writing – review and editing. **Tuhan Oruk:** Conceptualization; resources; writing – original draft. **Yavuz Selim Kıyak:** Conceptualization; methodology; writing – review and editing. **Tuncay Peker:** Conceptualization; data curation; formal analysis; funding acquisition; investigation; methodology; project administration; resources; software; supervision; validation; visualization; writing – original draft; writing – review and editing.

## CONFLICT OF INTEREST STATEMENT

There is no conflict of interest in the present form of the manuscript.

## Supporting information


Data S1.

Data S2.
