# Supplementary material for: A new classmate in anatomy education: 3D anatomical modeling medical students’ engagement on learning through self‐prepared anatomical models
Source: Anat Sci Educ. 2025 Jun 17;18(7):727–37. doi: 10.1002/ase.70070 (PMC12222579; doi:10.1002/ase.70070)
Supplement: Supplementary file 1 — Data S1. [file ASE-18-727-s001.pdf]

Below, a part of the instructions of the educator for the modelling of neurons (Figure 2) has been added:

We will create a neuron in this lesson.

We will start by choosing the object “platonic.” Then for this object, we select the “make editable” tool.

To create a neuron we will start with dendrites.

To mark the surfaces of the object that the dendrites would sprout, we select the “polygons” tool of the tools on the top.

To be able to mark more than one surface at a time we click “shift” as we mark them.

Then we right-click and select the “inset” tool.

With this option, we click on the mouse and slide the cursor to the left at the same time to minimize the surfaces.

After the first minimization, we make a second minor one. Then we right-click again and select the “matrix extrude” tool.

With this option, again we click on and slide the cursor to the right this time to sprout dendrites.

Now with some minor adjustments, we engage the courses of dendrites.

Now that the neuron body is ready, we can move on with the axon. To create the axon, we mark one surface of the initial object and apply the same moves to this one surface as we did for dendrites.

We are done with cell parts. Now to make adjustments to the cell as a whole we select the “model” option from the top menu.

Our neuron is completed.

Now to add color to the cell, we use the “material manager” tool which is at the right corner of the minor screen.

After we select this tool, we drag the material (“Mat”) we just created to the right list and drop it under the object “Platonic.”

We double-click the material (“Mat”) and open the “material editor” window. In this window, we add a texture to the object.

To observe the texture and colour we add, we view the rendered image of the object.

We make the adjustments that are shown in the video for texture and colour. With these adjustments, we will animate an impulse that goes through the neuron.
