# Supplementary material for: A new classmate in anatomy education: 3D anatomical modeling medical students’ engagement on learning through self‐prepared anatomical models
Source: Anat Sci Educ. 2025 Jun 17;18(7):727–37. doi: 10.1002/ase.70070 (PMC12222579; doi:10.1002/ase.70070)
Supplement: Supplementary file 2 — Data S2. [file ASE-18-727-s002.pdf]

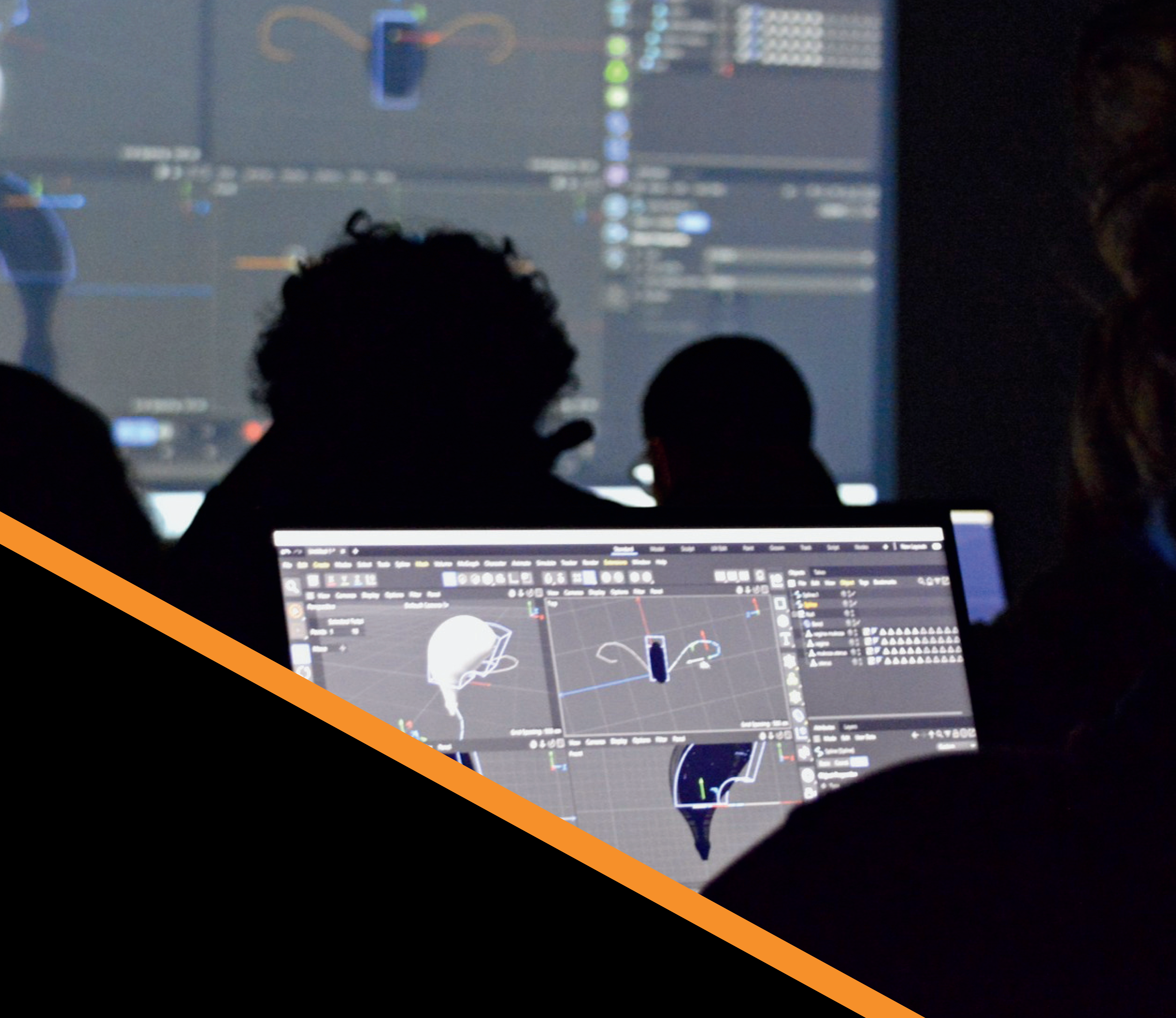

UNDER THE GUIDANCE OF
PROF. DR. TUNCAY PEKER

3D ANATOMICAL MODELING WORKSHOP BOOKLET

MAXON CINEMA 4D

Study Group:

Afife Zehra YURTSEVER
Muhiddin Furkan KILIÇ
Ezgihan ERTUÇ
Tuhan ORUK
Beste BAŞGUT
Feyza AÇIKGÖZ
Burcu MAVİ
Sinem SEVİM
Yavuz Selim Kıyak
Tuncay Veysel Peker

CONTENTS

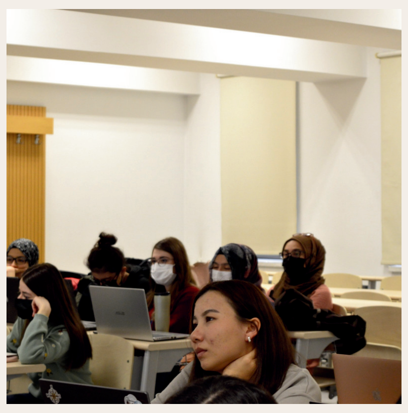

1 About Us
PAGE 2

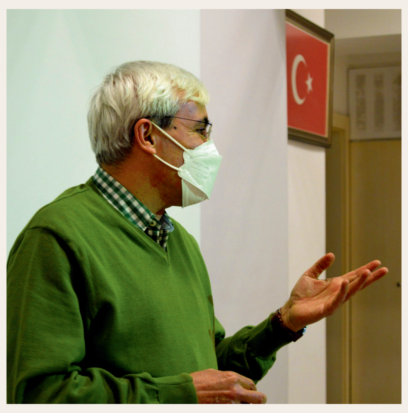

2 Who is Tuncay Peker?
PAGE 3-4

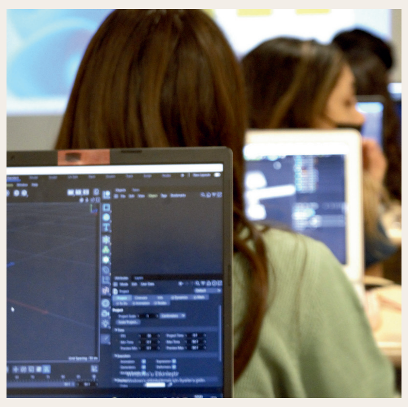

3 Our Curriculum
PAGE 5-7

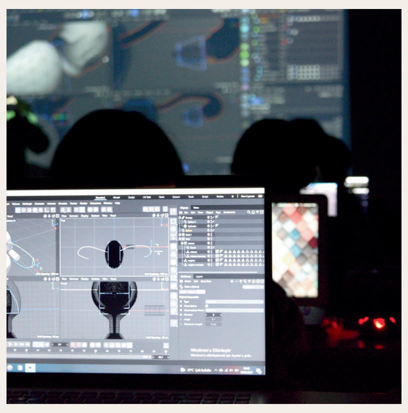

4 Learning Outcomes
PAGE 9

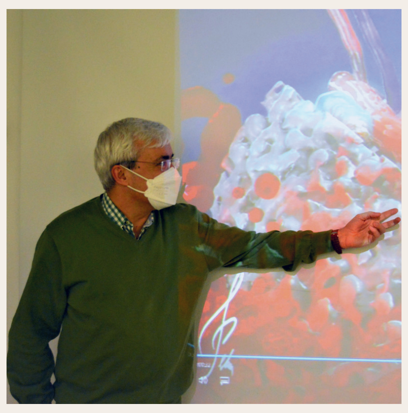

5 From the Professor's Perspective
PAGE 10

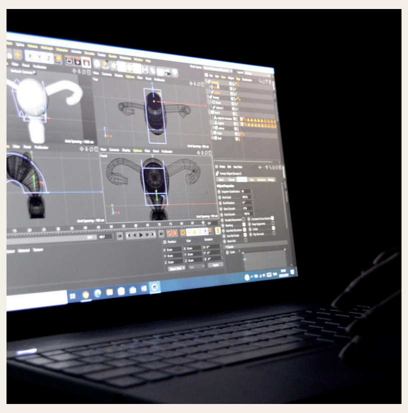

6 Our Work
PAGE 10-12

ABOUT US...

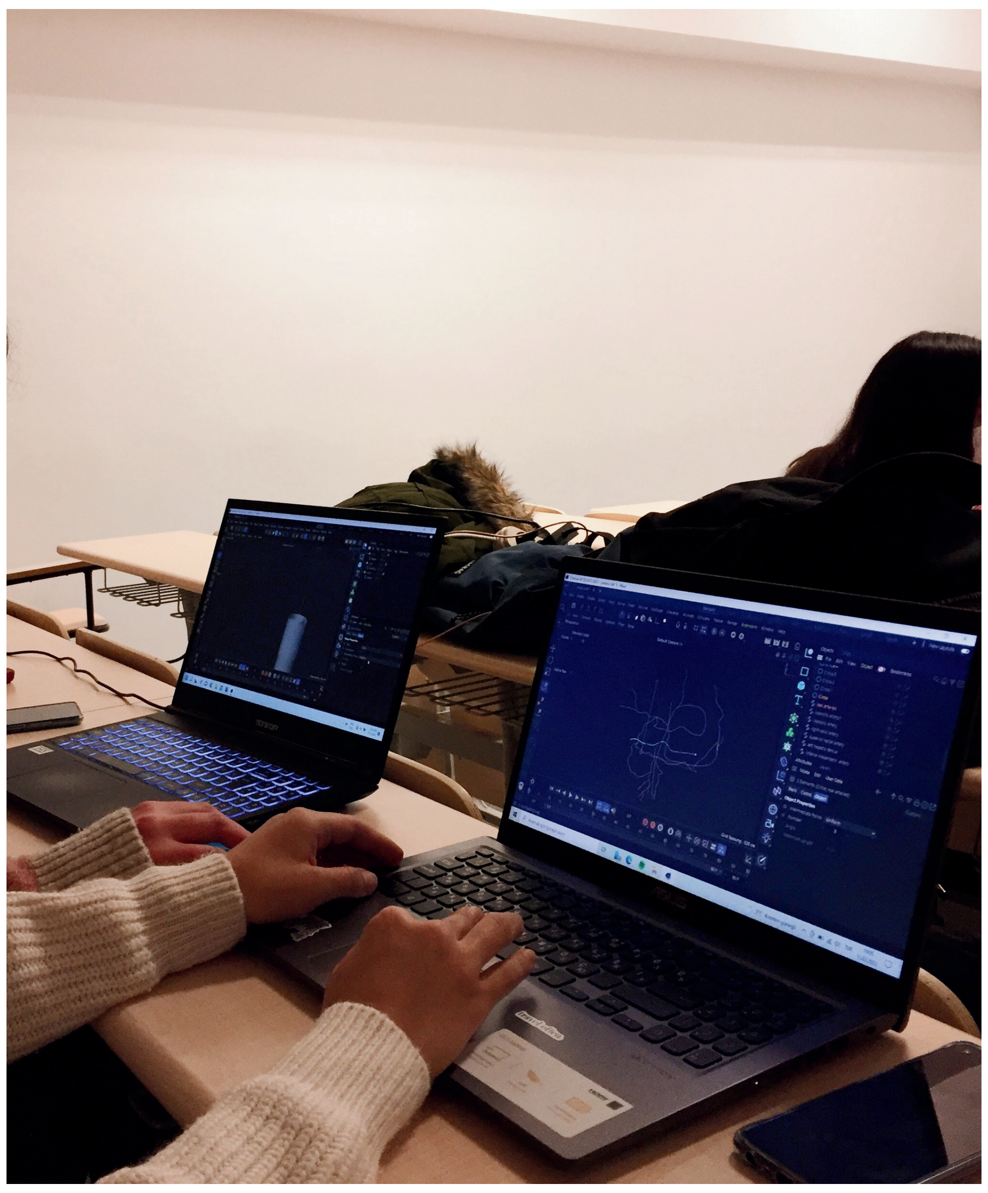

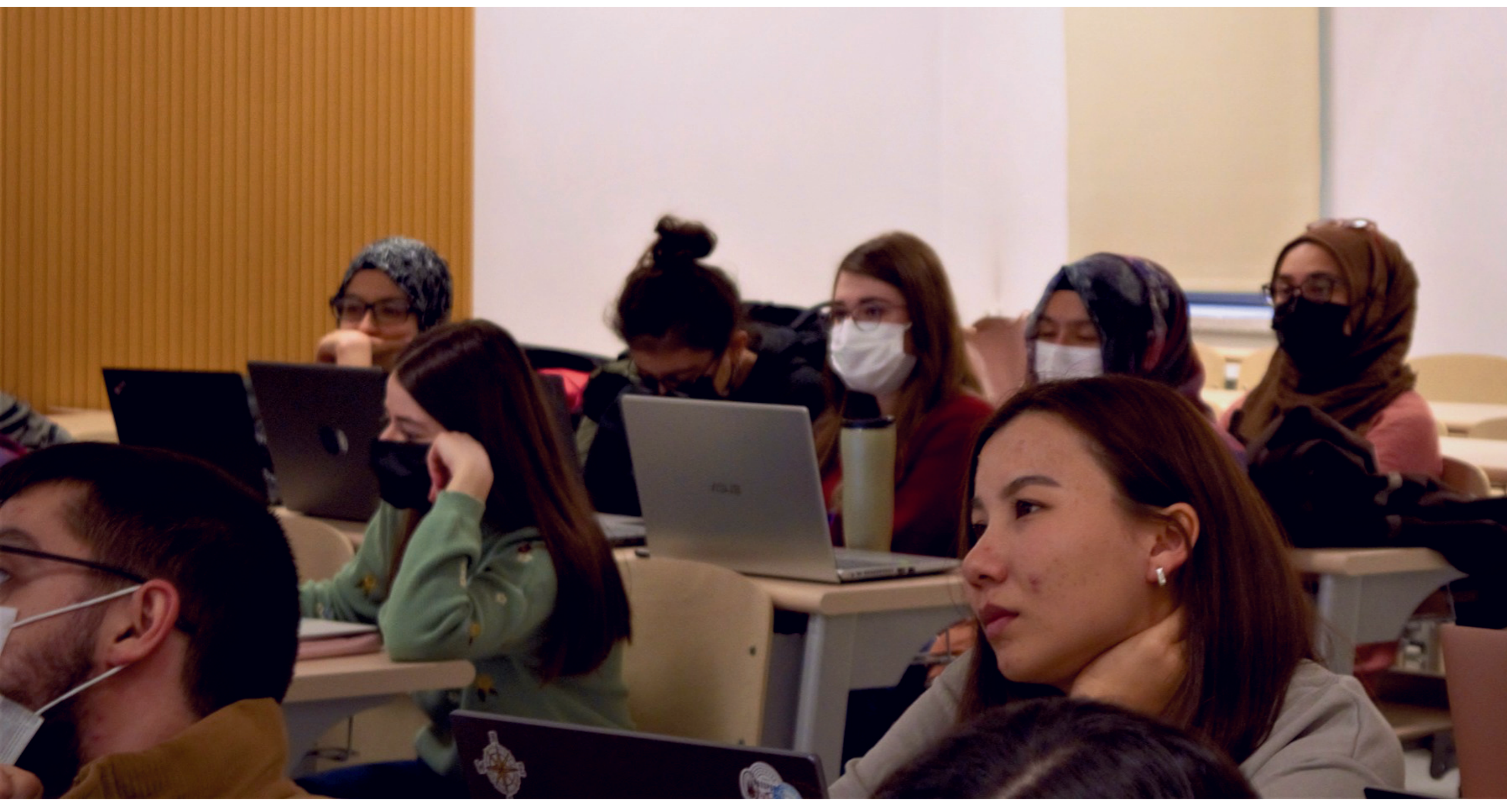

Our team, consisting of students from the Faculty of Medicine at Gazi University, used Maxon Cinema 4D for the training.

Our lectures progressed synchronously under the leadership of Prof. Dr. Tuncay V. Peker. The participation of students with different levels of knowledge has shaped the workshop's progression and facilitated communication among them. In addition, the continuity of the course was maintained with weekly assignments, and students' work was assessed through feedback.

ABOUT TUNCAY PEKER

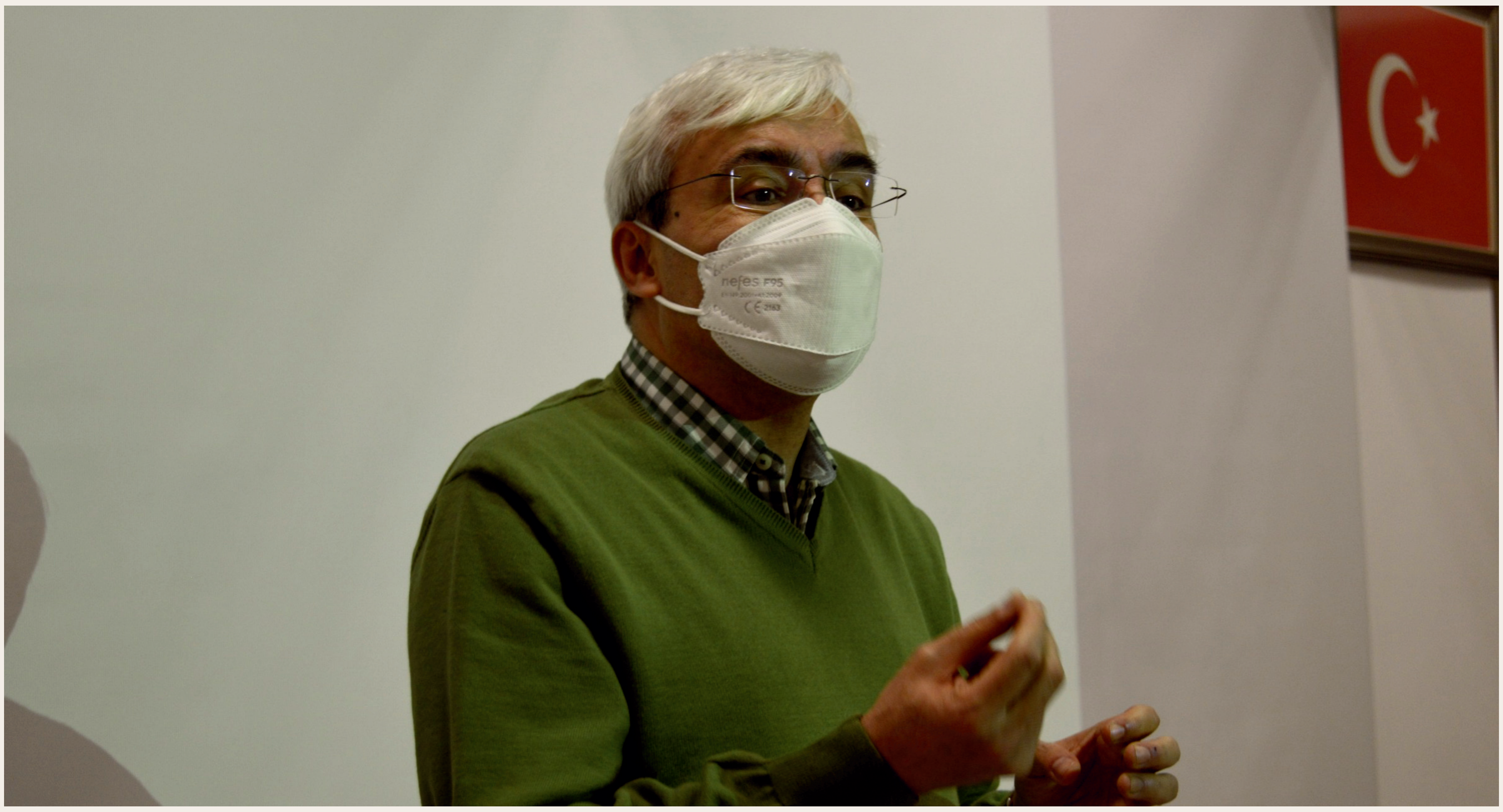

Tuncay Peker was born on June 2nd, 1962, in Ankara. After completing his primary, secondary, and high school education in Ankara, he began his medical studies at the Faculty of Medicine at Ankara University, graduating in 1987. In 1991, he started working as an assistant in the Anatomy Department at the Faculty of Medicine, Gazi University. He became an Anatomy Specialist in 1993, an Associate Professor in 1998, and a Professor in 2003. He is currently working as a faculty member at the same institution.

WHO IS TUNCAY PEKER?

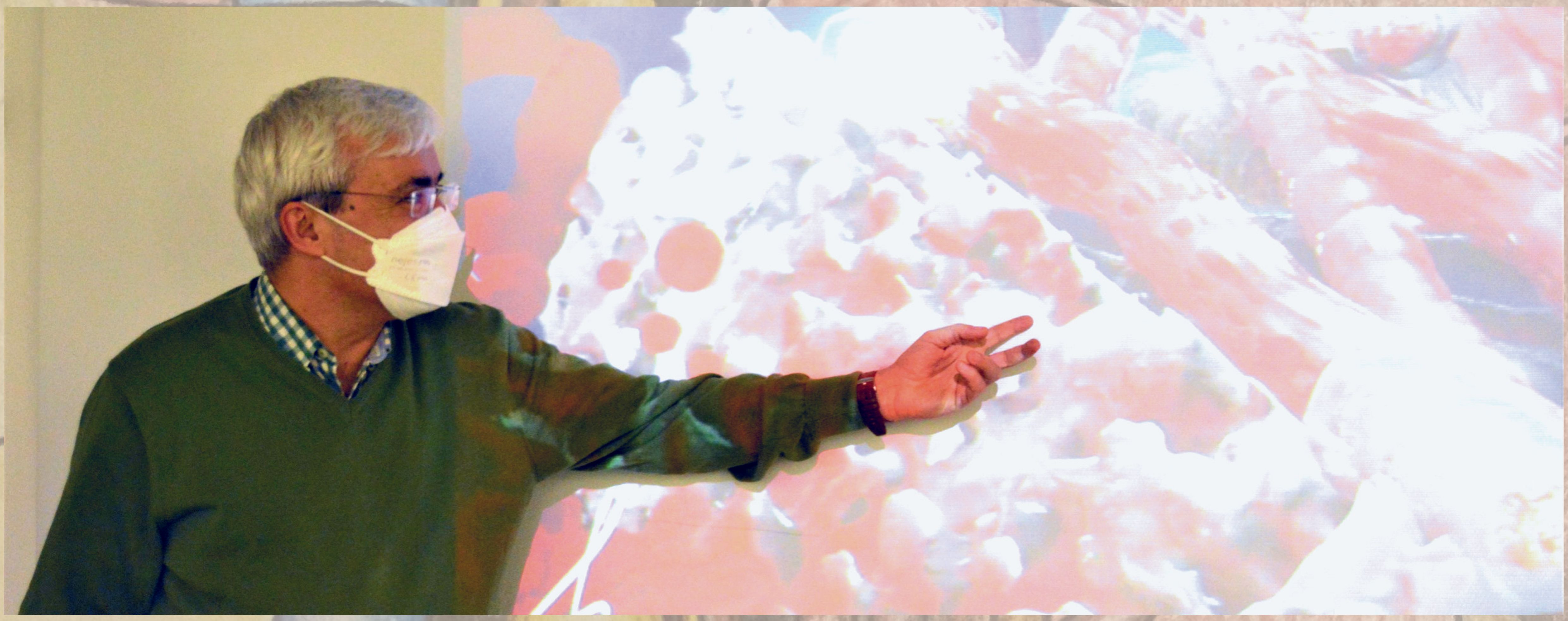

Tuncay V. Peker, who has been interested in drawing since childhood, began creating anatomical drawings after entering medical school. With a desire to integrate 4D technology into his passion for drawing, he started his studies in 2008, teaching himself Maxon Cinema 4D. As a medical doctor and academic, Peker's goal is to create 3D medical modeling projects and incorporate them into education. In December 2021, the professor was awarded the Innovator Medical Doctor of Basic Science of the Year Award in Turkey.

The modeling workshop started with a general introduction to medical modeling and the history of VR/AR screening. In the beginning, we discussed the general functions of Maxon Cinema 4D and created an erythrocyte model using basic shapes. Later on, using functions like segmentation, cloning, and coloring, we modeled the movement of an erythrocyte inside a vessel. Lastly, by adding a macrophage to the model, we completed a basic animation of blood cells.

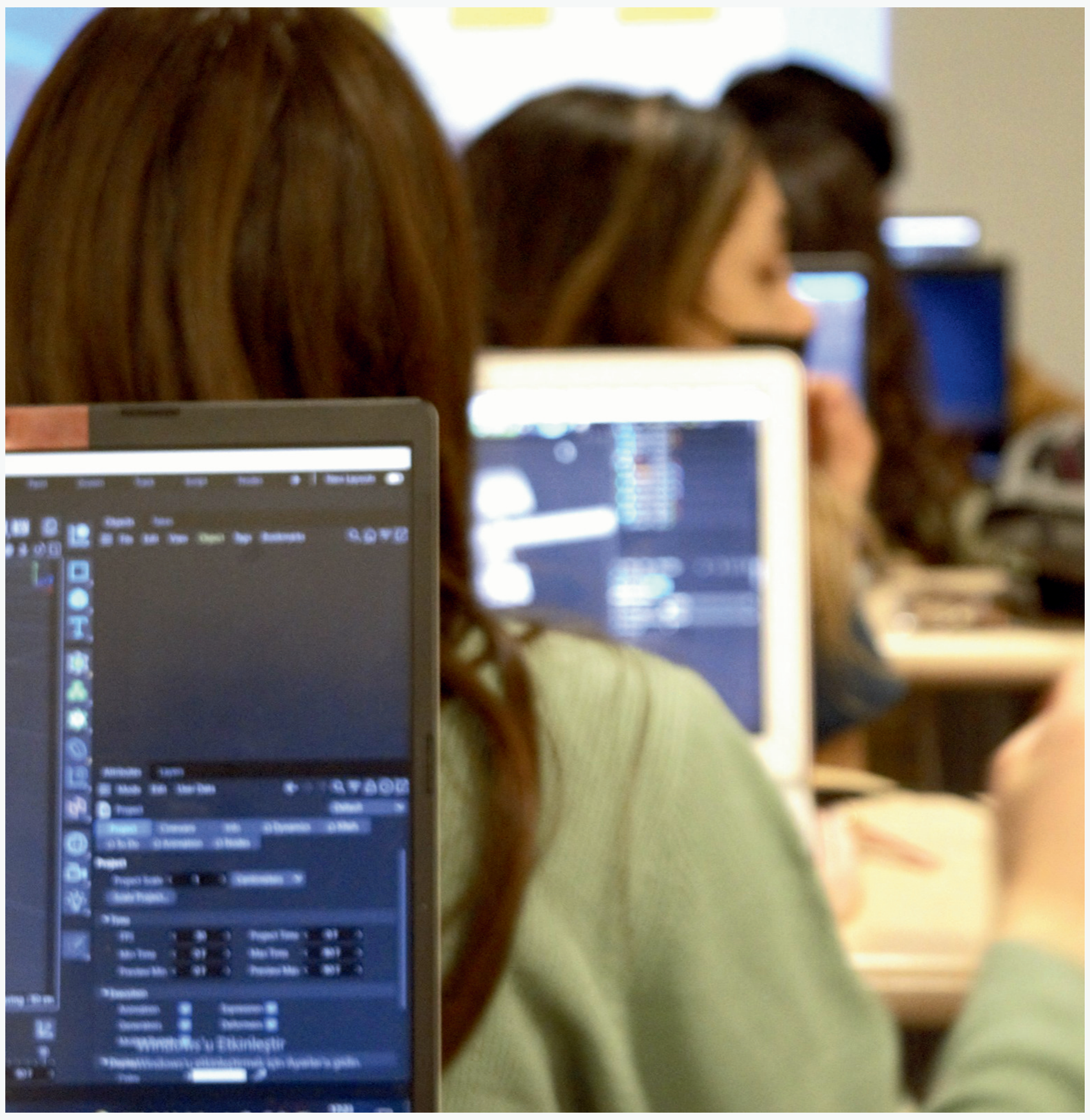

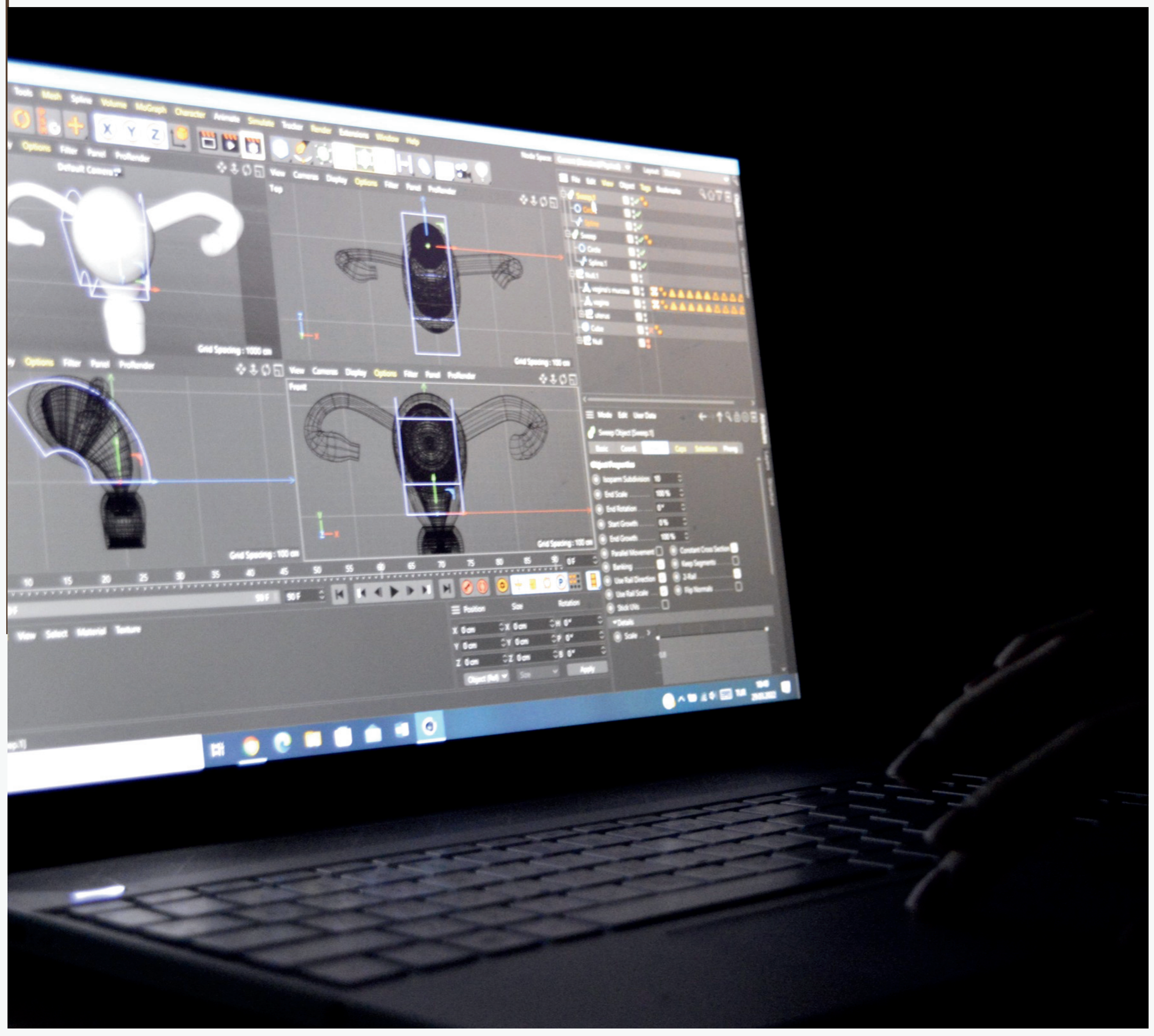

In the following weeks of the workshop, we learned new functions such as fragmentation, tearing, and shrinking on objects, and we used these functions to create an animation of an 'apoptotic cell.' In addition to this, by adding apical structuring to a cell, we created a 'ciliated epithelial cell' and a 'basic neuron model.

OUR CURRIUCULUM

- In another lesson, we used various functions to study the anatomical positions of intra-abdominal organs and the vascular structures of the upper abdomen. We modeled structures such as the pancreas, duodenum, gallbladder, kidneys, and adrenal glands. We also shaped a hollow structure to resemble a stomach and created the stomach contents by deforming spherical shapes. Finally, we generated a 'food passage through the stomach' model.

- We also learned how to apply a rough texture to the bone tissue to create a tissue microenvironment. Then, we used this microenvironment for the 'bacterial phagocytosis animation.

- In a different lesson, we drew the intraocular structures, including the cornea, sclera, choroid layer, and lens, in 2D. Then, we converted them into 3D using Maxon Cinema 4D functions. In addition to this, we created a surgical instrument using cubes and cylinders and developed a 'cataract surgery' model.

- As a project, we first drew the arterial wall structures in 2D and then converted them into 3D using Maxon Cinema 4D functions. We took a cross-section of the modeled vessel to make its interior visible and illustrated the atherosclerotic plaque structure within the opened section. After that, we modeled a stent and placed it on the vessel wall using a balloon, creating a 'stent placement' animation.

- As another project, we first created the shape and mucosa of the uterus, then drew and positioned the Fallopian tubes. We placed fimbriae structures at the ends of the Fallopian tubes. After that, we used spherical shapes to draw the ovaries at the fimbriae ends and modeled the 'general structure of the female genital organs.

OUR CURRICULUM

LEARNING OUTCOMES

Evaluation Aspect	Mean±SD	Median (min-max)
Rating for content	8,24±1,35	8,00(5-10)
Contribution of the workshop to medical lectures	5,66±2,25	6,00(2-10)
Recalling the features of the program used after the workshop	6,90±1,95	7,00(2-10)
Request for 3D printout from the 3D modeled structure	9,45±1,18	10,00(6-10)
Rate of reduction of stress and anxiety towards classes	6,24±2,11	7,00(1-10)
Rate of using workshop knowledge in future professional life	8,69±1,44	9,00(5-10)
Coordination of students with the instructor	7,66±1,95	7,00(3-10)
Adequacy of the instructor's knowledge	9,48±0,78	10,00(7-10)
The instructor's explanation is clear and understandable	9,07±1,06	9,00(7-10)

Table. Participant ratings of the anatomical modeling workshop based on a 1 (low) –10 (high) scale.

- Looking at the overall course, students have a basic understanding of the functions used in 3D modeling programs. Although it may sound like a cliché, this course demonstrates that imagination has no limits. In the world of modeling, there is no single way to draw a simple object or figure from one's mind. We can confidently say that the only requirement for creating any shape is simply to think.

- The course which is an introduction to 4D modelling technology, provides the students to put an effort in a field other than medicine.

- The modeling of anatomical visuals by students reinforces the lessons. In fact, it has also made it easier for first-year students who have not yet started anatomy classes to integrate into the course.

FROM THE PROFESSOR'S PERSPECTIVE

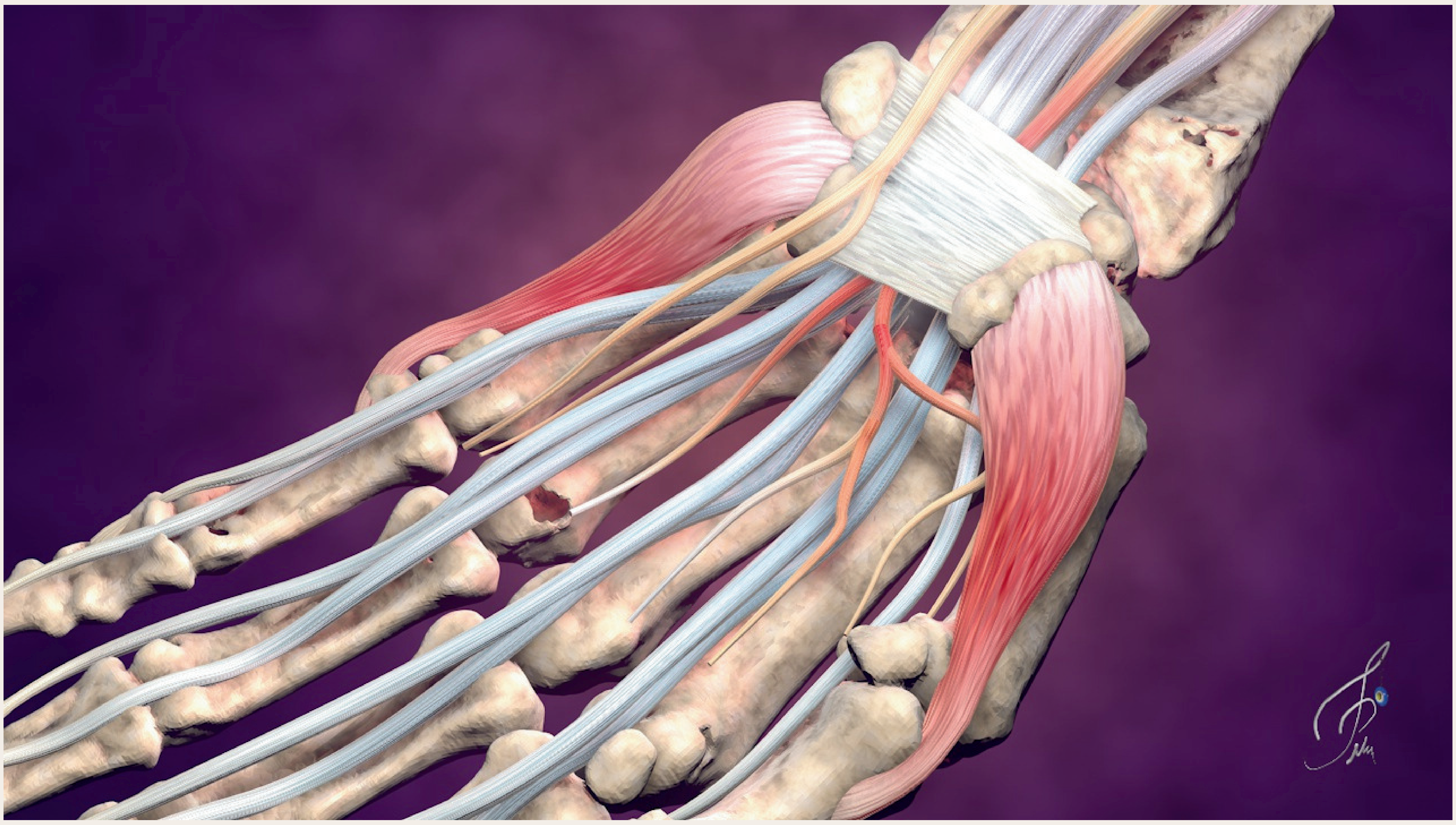

Tuncay Veysel Peker

Did the training satisfy your expectations?

It satisfied my expectations perfectly. It was even beyond my expectations. It made me so happy. Students were so interested, and their high expectations motivated me. In time we began to do much better work.

What do you think about the performances of the students who participated in the course?

They were incredibly good. I even thought that they were faster than me with several things. They came up with some things that I couldn't think of.

What are your plans for modeling for the later periods?

I am eager to make a VR anatomy module, and I'm planning on finishing it. Completing it will allow me to leave a lasting impact on the world

What was your favorite thing about the modeling training?

Students' interests and enthusiasm made me so happy.

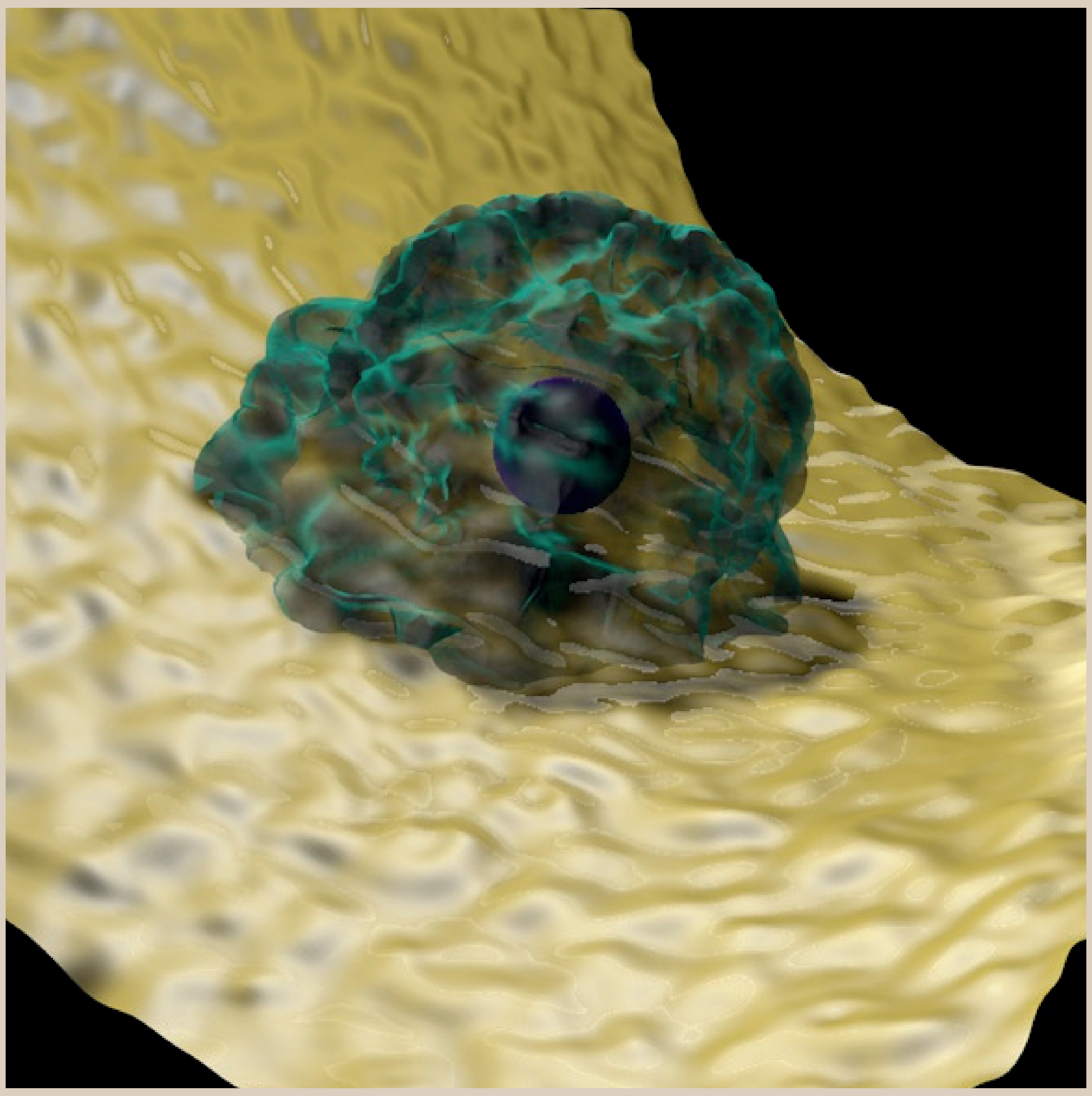

Beste Başgut

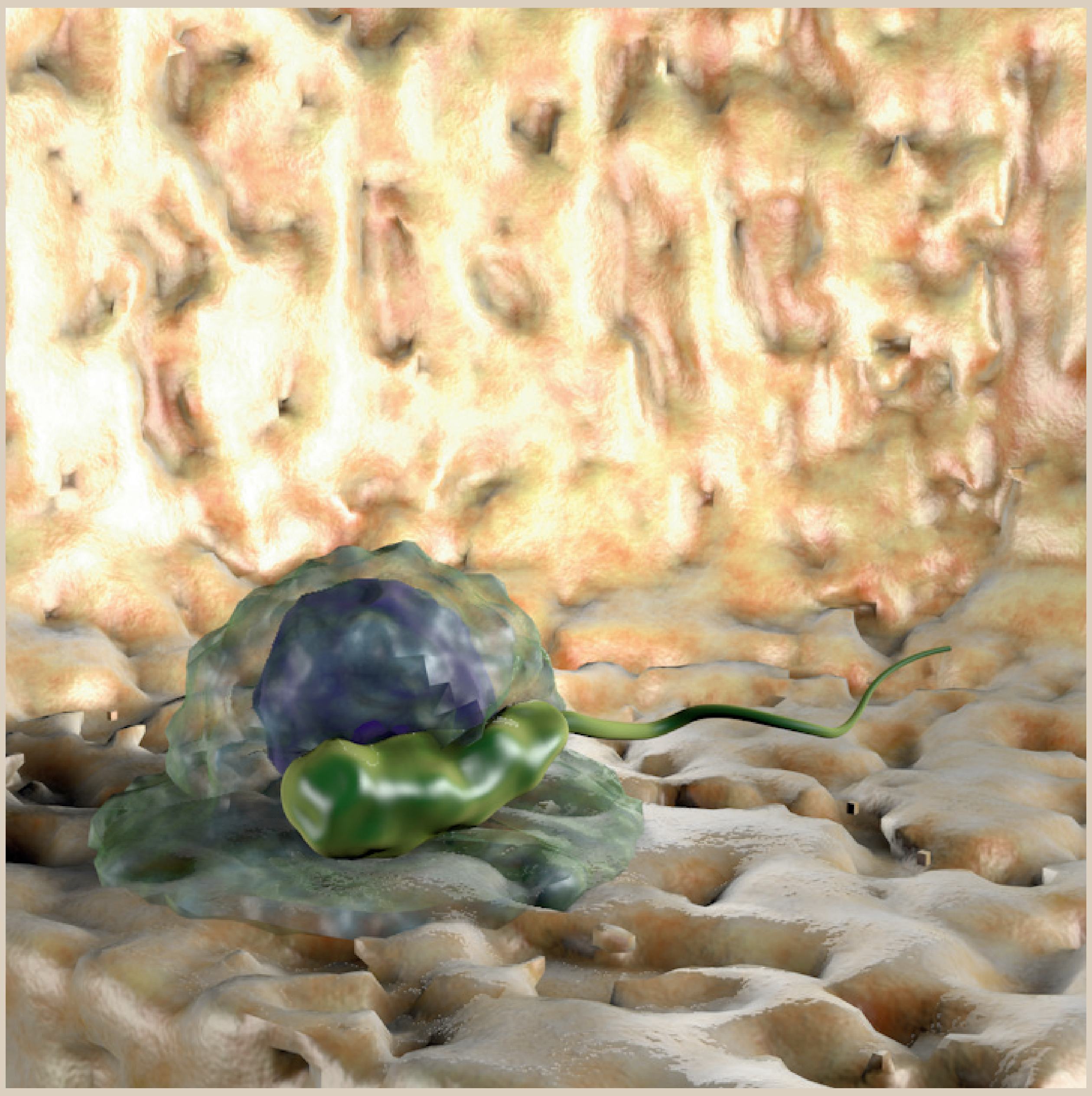

Ömer Tuna Çiçekdal

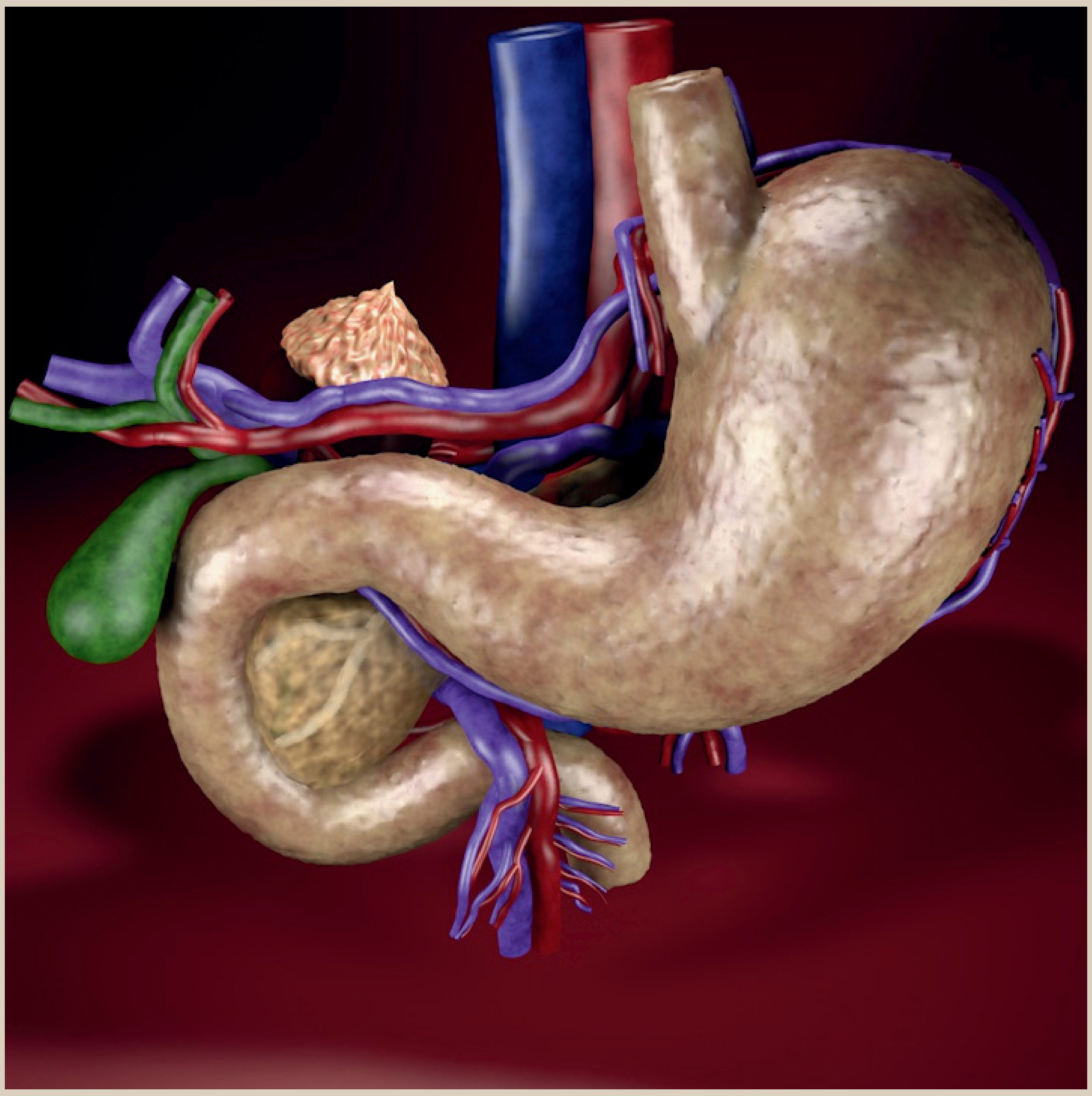

Beyzanur Koç

OUR WORK ↑

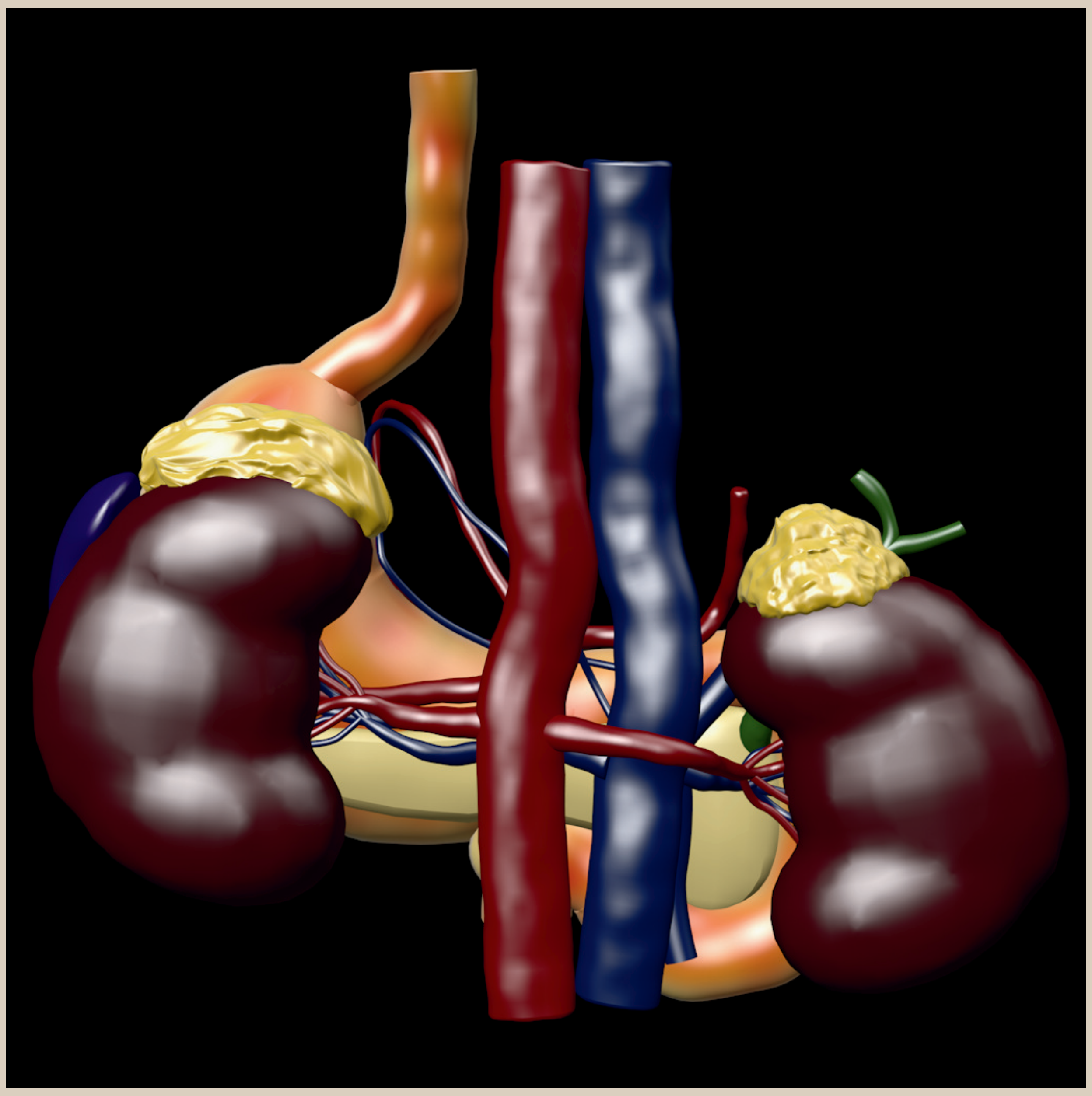

Halime Melike Öncü

"Thanks to the workshop, I gained skills in 3D design and animation. The benefits of the workshop weren’t one-dimensional; it also helped me academically. The projects we worked on alongside our topics, such as organs, vessels, nerves, and other structures, made it much easier for me to visualize their anatomical localization and helped make them more memorable."
Halime Melike ÖNCÜ

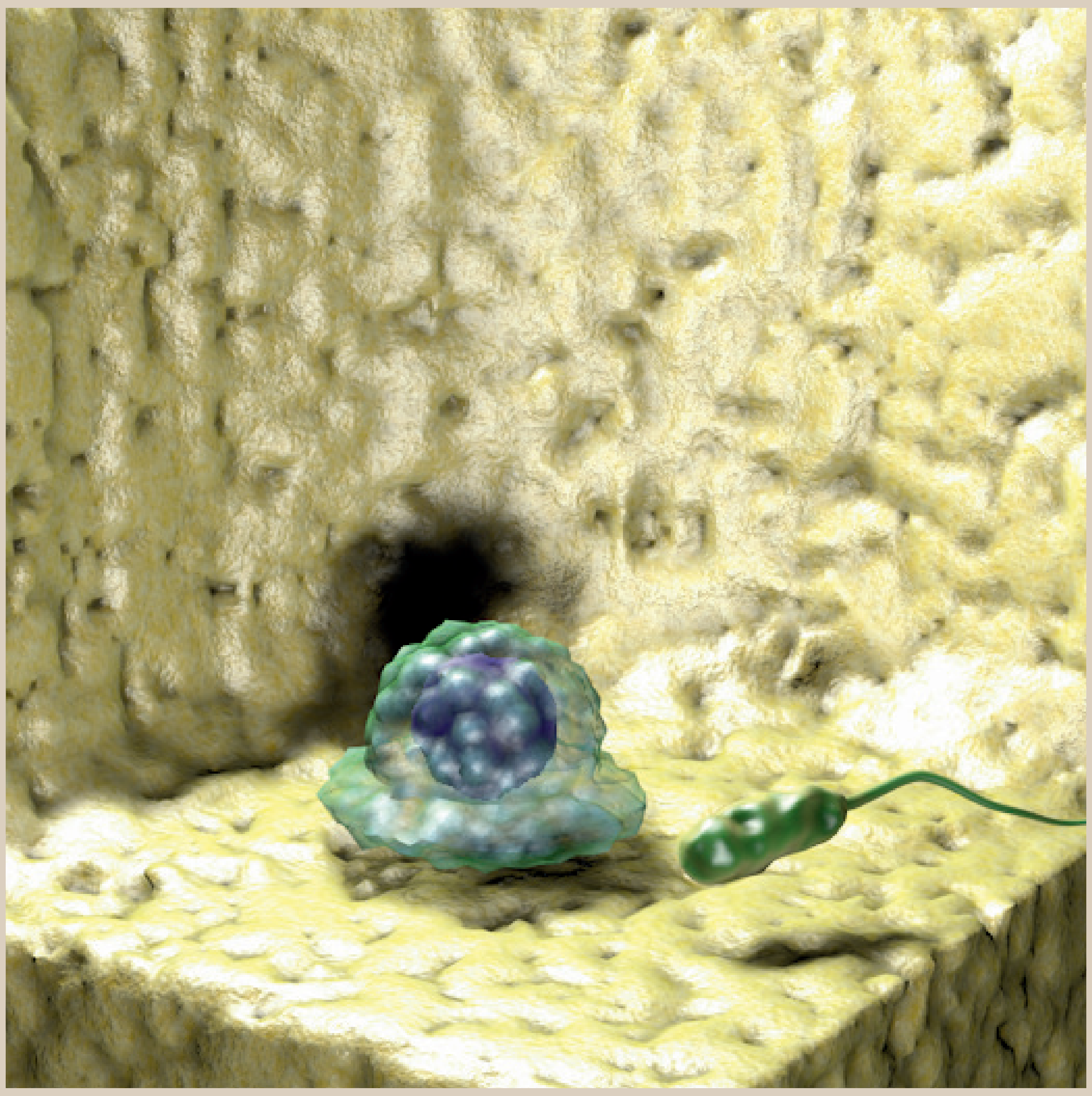

Sinem Sevim

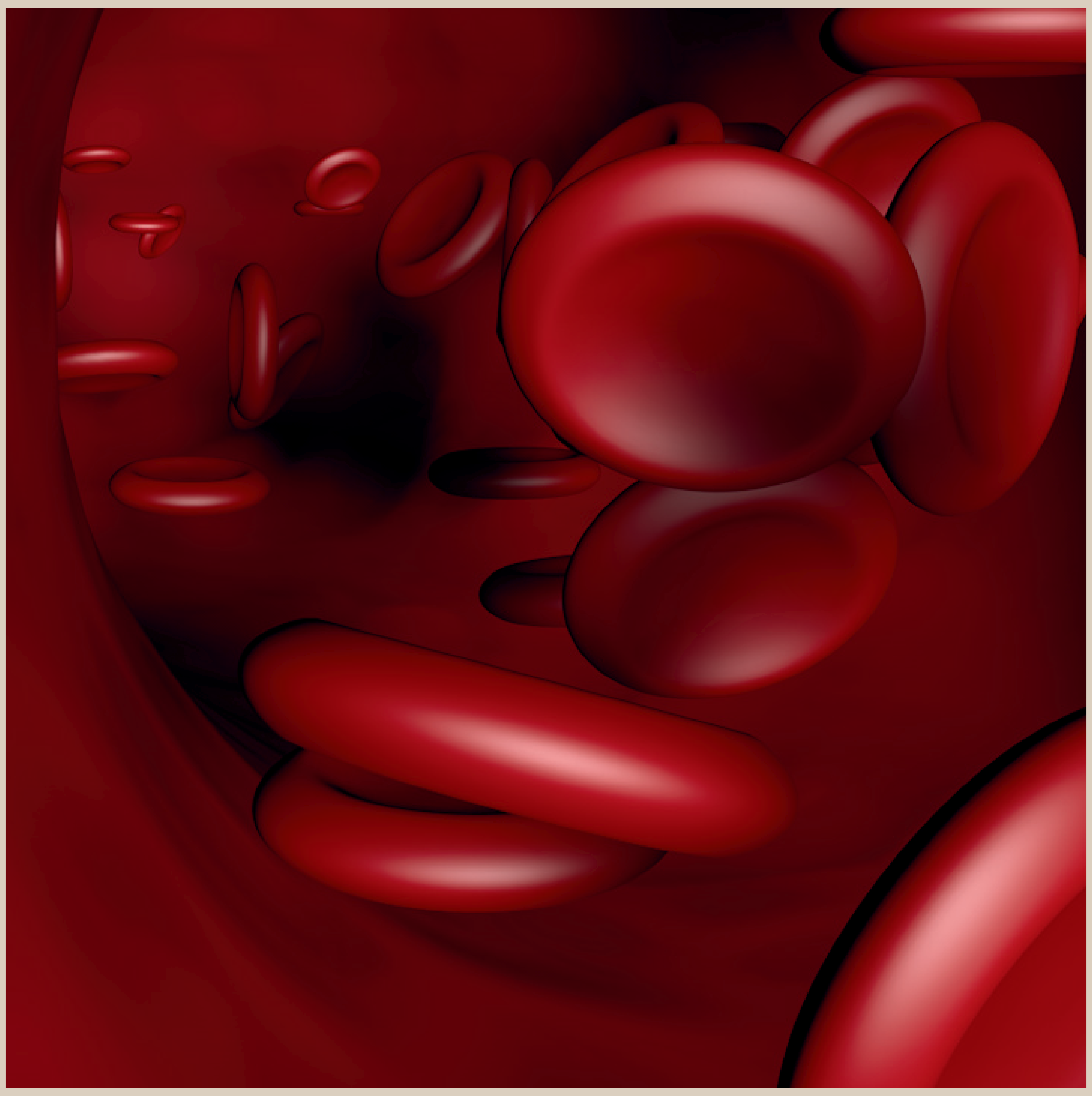

Afife Zehra Yurtsever

OUR WORK ↑

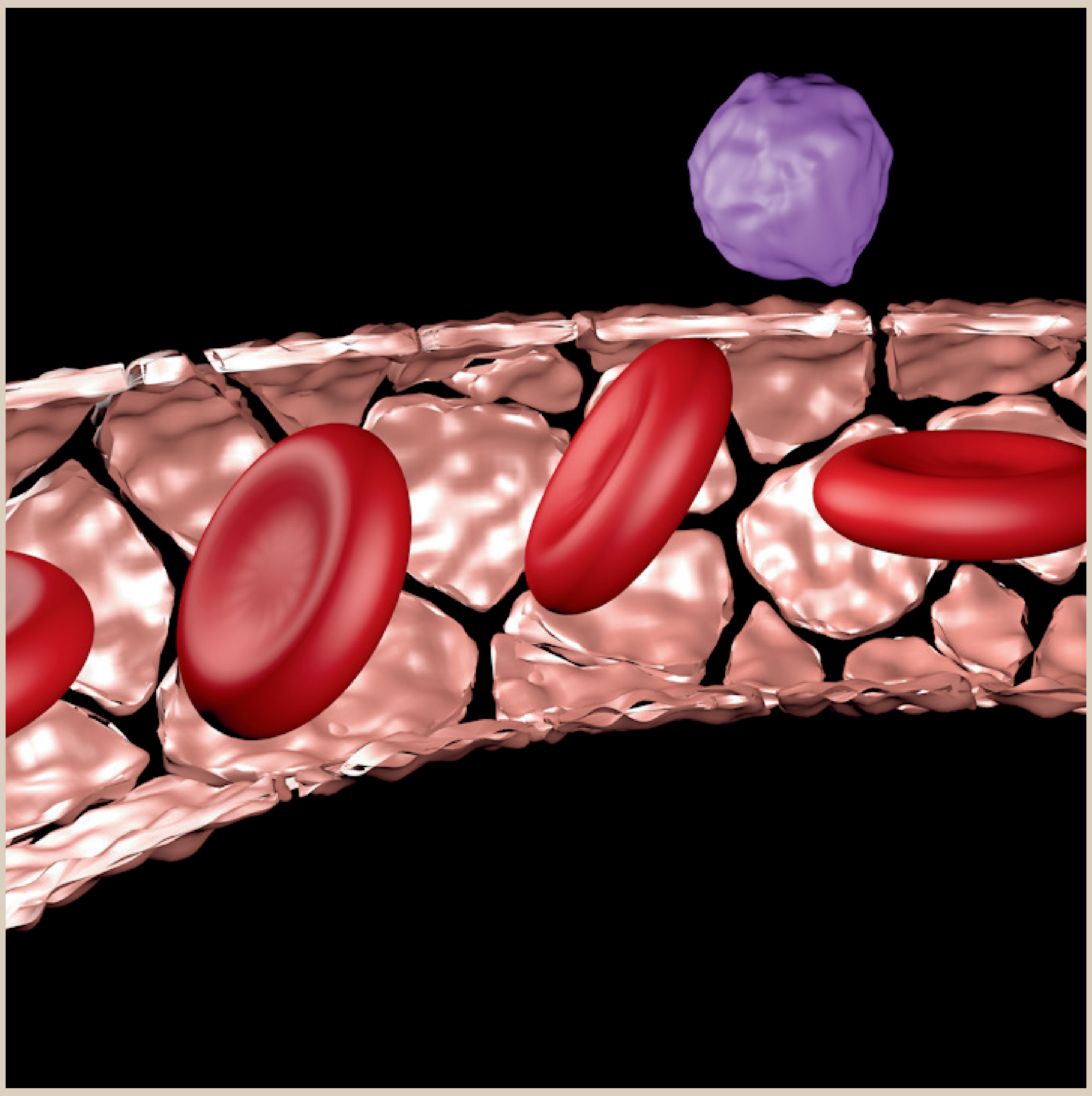

Asiye İlayda Büyükgebiz

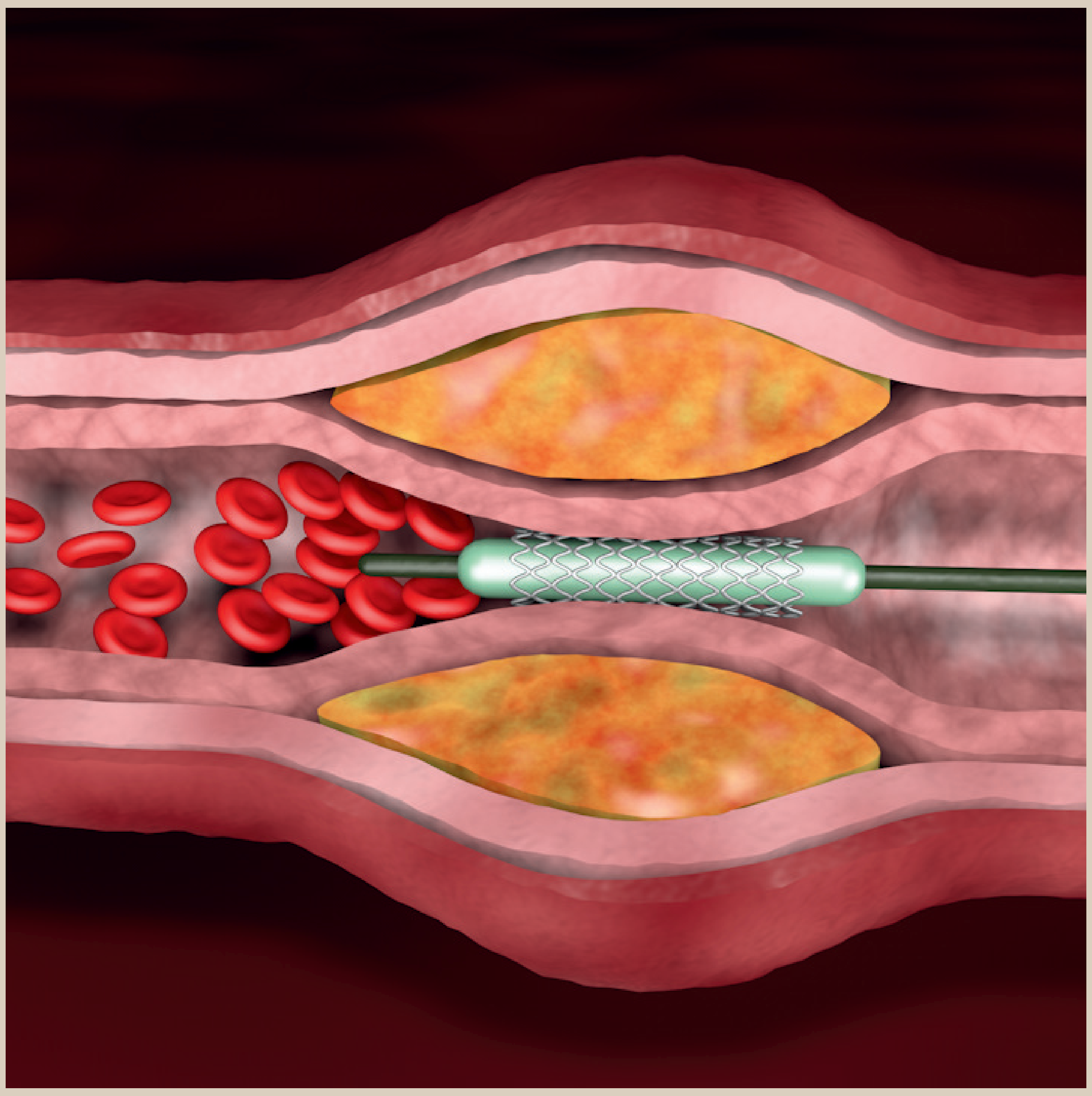

Beyzanur Koç

"I learned to look at things from different perspectives because, in the workshop, we can create a model in multiple ways. How and what we create is entirely up to us and our imagination."
Burcu MAVİ

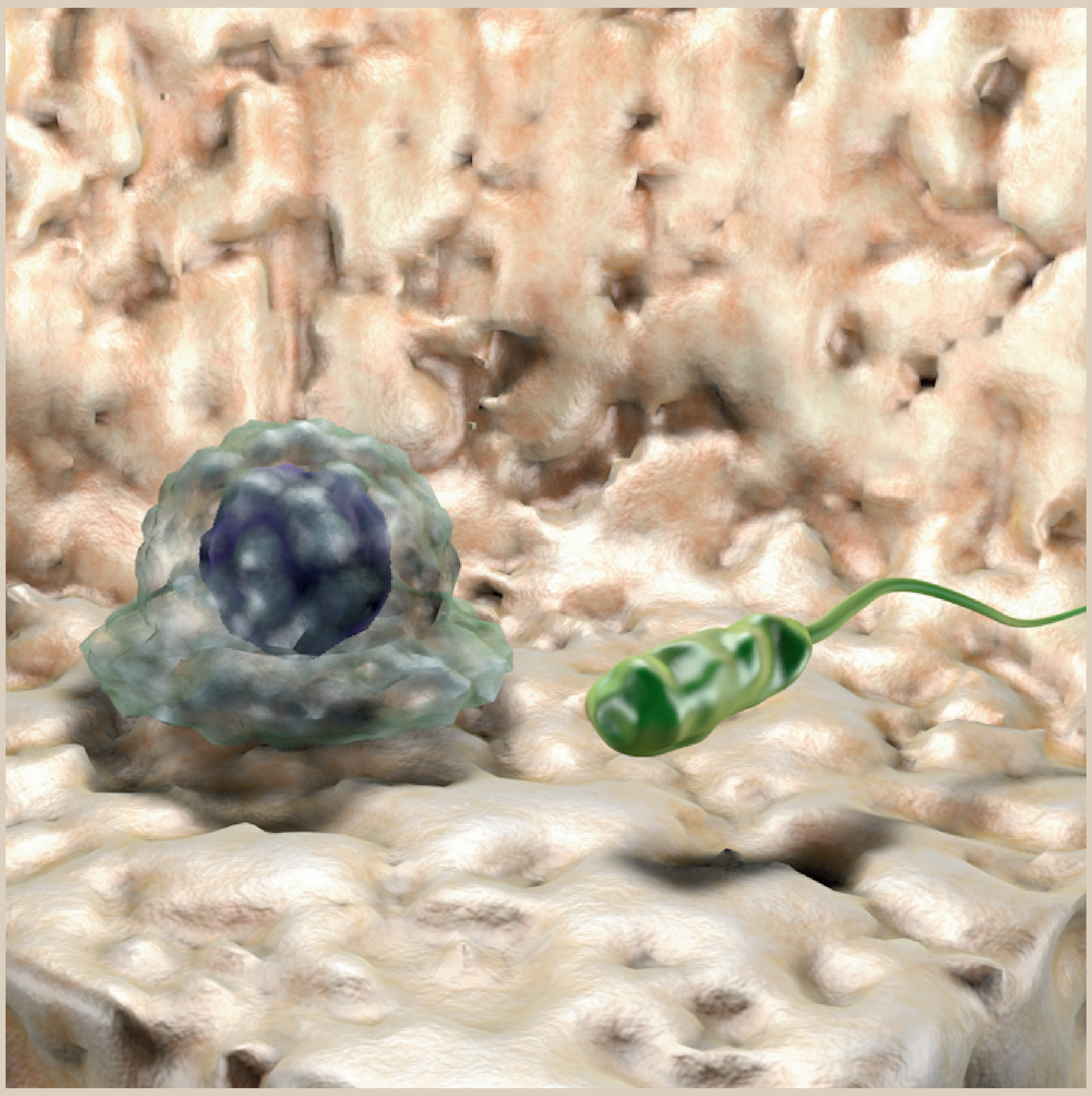

Burcu Mavi

OUR WORK ↑

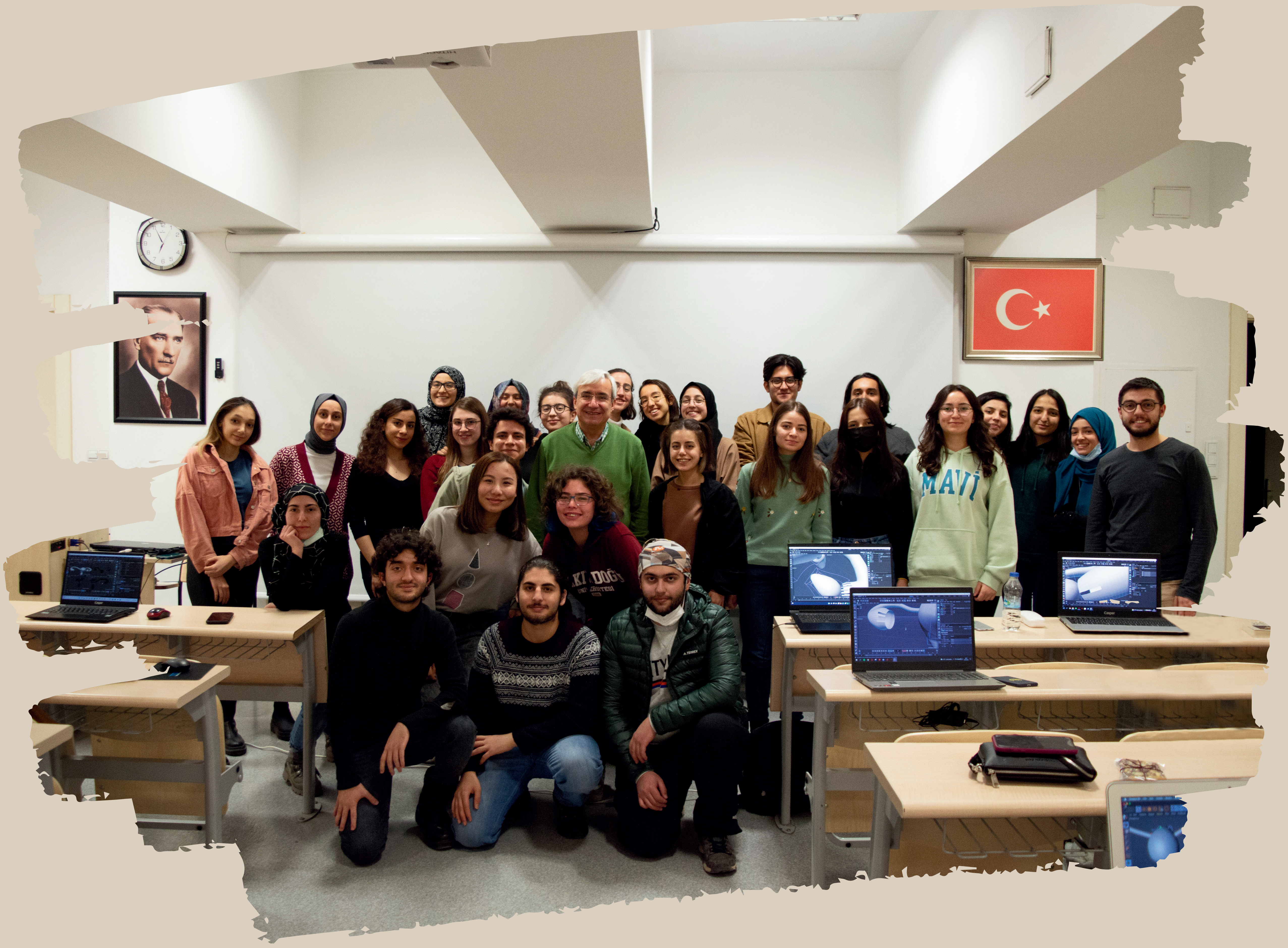

“ From Anatomical Modeling Workshop
Prof. Dr. Tuncay Peker and His Students ”

Faculty of Medicine
Gazi University
2022
